# High-fidelity avatars for behavior and cognition research in virtual environments: An accessible construction method for scientists

**DOI:** 10.3758/s13428-026-03139-1

**Published:** 2026-08-19

**Authors:** Rachael L. Taylor, Lisa Huerta, A. Mike Burton, Markus Bindemann

**Affiliations:** 1 School of Psychology, University of Kent, Canterbury, UK; 2 Department of Psychology, University of York, York, UK; 3 Faculty of Society and Design, Bond University, Gold Coast, Australia

**Keywords:** Virtual reality, Avatar, Face perception, Gaze cueing, Emotional expression

## Abstract

Virtual reality is increasingly employed in behavioral research, providing immersive environments that combine high levels of experimental control with ecological validity. Within virtual reality, humans are represented by digital characters - or avatars - whose realism and behavioral fidelity are crucial for studying social cognition. This study introduces an accessible method for constructing avatars with facial animations of eye gaze and expression that can be used by behavioral scientists. In two pre-registered experiments, we validate the use of these avatars for psychological research. In Experiment 1, we demonstrate that observers orient attention in response to avatar eye gaze, replicating the classic gaze-cueing effect. In Experiment 2, we show that participants can recognize avatar facial expressions with accuracy and confusion patterns comparable to those reported for human faces. These findings establish our construction pipeline as a user-friendly approach for generating high-fidelity avatars, equipping researchers with tools to investigate social and cognitive processes in controlled yet ecologically valid virtual environments. We provide access to an extensive manual for constructing avatars, along with a video example demonstrating the procedure step-by-step. Using a relatively inexpensive hand-held scanner, this enables behavioral scientists to create avatars with accurate facial animation for experiments in virtual environments.

**Supplementary Information:**

The online version contains supplementary material available at https://doi.org/10.3758/s13428-026-03139-1.

## Introduction

Virtual reality is becoming widely adopted as a tool in behavioral research. The availability of affordable, high-quality head-mounted displays (HMDs) has been a key driver of this surge (Wilson & Soranzo, 2015; Vasser & Aru, 2020). Beyond accessibility, virtual reality provides unique methodological advantages. It enables researchers to construct immersive, visually complex environments in which increasingly realistic behavior can be studied under conditions of high experimental control (Fysh et al., 2022; Loomis et al., 1999.

Faces represent one of the most frequently used visual stimuli in behavioral science, yet traditional methods typically rely on static, two-dimensional photographs presented without meaningful context. This contrasts with everyday social interaction, where faces are dynamic, three-dimensional, and embedded within broader interpersonal contexts. While studies employing live confederates provide greater ecological validity, they often lack the precision and control required to isolate specific cognitive mechanisms. Virtual reality can serve as a compromise between the highly controlled nature of laboratory studies and the greater ecological validity of field research by combining experimental control with immersive realism.

To study person perception in virtual reality, digital humans - or *avatars* - may be inserted into virtual environments. Avatars can be designed to perform socially relevant behaviors such as gesturing and posturing (Etienne et al., 2023), displaying facial expressions (Geraets et al., 2021; Kimmel et al., 2023), and directing eye gaze (Garau et al., 2001; Garau et al., 2003; Zheleva et al., 2023). Although state-of-the-art game engines demonstrate that avatars can achieve impressive levels of visual and behavioral realism (see, e.g., ‘Enemies’, Unity Technologies, 2023; ‘The Heretic: Digital Human’, Unity Technologies, 2020), such productions typically require large teams of skilled 3D artists, riggers, and animators, alongside access to specialized software. This level of expertise and resourcing is beyond the reach of many behavioral scientists, highlighting the need for accessible methods of constructing high-fidelity avatars.

Several approaches exist for producing avatars resembling real individuals. For example, researchers have mapped photographs onto 3D meshes or generated 3D avatars from multiple 2D images (Bailenson et al., 2008; Grewe et al., 2021; Tummon et al., 2019). However, these methods often inadequately capture critical facial texture and geometry (Fysh et al., 2022), both of which are fundamental for recognition (Burton et al., 2005; Hole et al., 2002; Rogers et al., 2022; Caharel et al., 2009; O’Toole et al., 1999). Three-dimensional scanning provides a more accurate alternative, producing high-resolution measurements of facial structure (Bailenson et al., 2004). We have recently created an avatar construction method that utilizes such 3D scanning (Fysh et al., 2022). This method uses a photorealistic face scan, which captures the subject’s head geometry and texture. This scan is attached to a body, which is rigged for animation using free online software. Converging validation methods based on tests of face recognition, identity matching, and face-space analysis demonstrate that this approach captures the appearance of real people well (Fysh et al., 2022).

A limitation of this pipeline, however, is that avatars are restricted to static, neutral facial expressions. Face processing research spans beyond face recognition, encompassing domains such as attentional orienting to eye gaze (Bayliss et al., 2004; Driver et al., 1999; Friesen & Kingstone, 1998), emotion recognition (Lawrence et al., 2015; Zhou & Jenkins, 2020), and gaze-expression interactions (Adams et al., 2003; Adams & Kleck, 2005; Bindemann et al., 2008). Thus, manipulating eye gaze and emotional expression are important next steps to improve our avatar construction pipeline for wider use in research.

In this paper, we extend our accessible avatar construction method (e.g., Fysh et al., 2022) to incorporate eye gaze and emotional expressions. Our pipeline employs handheld 3D scanning and widely available software (including free academic tools) to produce avatars that are rigged for gaze and facial expression manipulation. A detailed construction manual accompanies this article to facilitate adoption by other researchers. Using this method, we generate example avatars that are validated in two experiments. These examined attentional orienting to gaze cues (Experiment 1) and tested recognition of basic emotional expressions (Experiment 2).

### Avatar construction

**Fig. 1:**
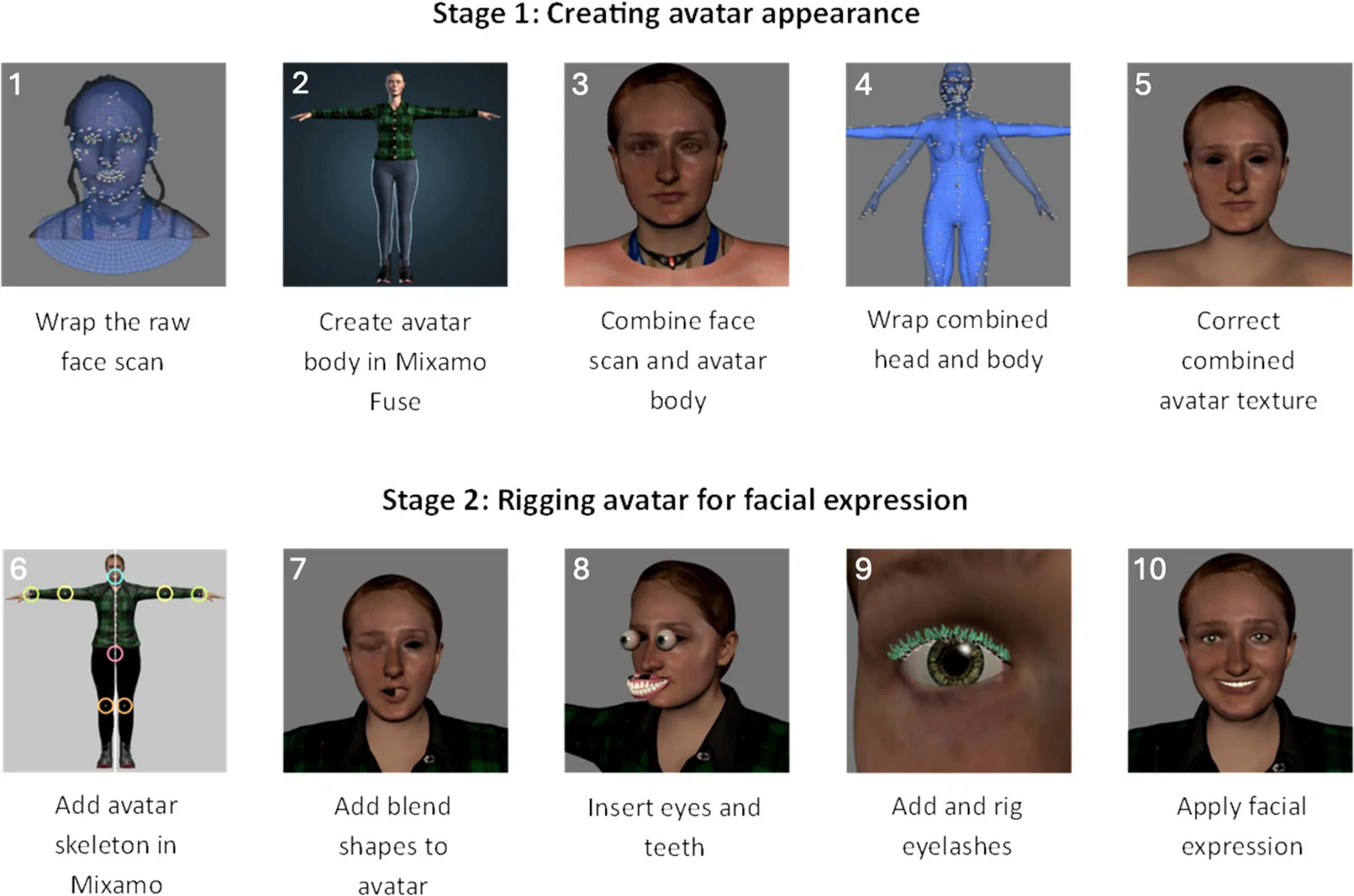
Overview of the construction pipeline, listing the steps to create avatars with animated eye gaze and facial expression

We summarize the key processes to create a high-fidelity avatar of a real person here. First, a 3D scan of a target person is acquired, following a procedure developed and validated in our previous research (Fysh et al., 2022). For this purpose, a person’s head is scanned from multiple angles while they maintain a neutral facial expression (using a handheld Artec Eva scanner and Artec Studio 15 software: Artec 3D, 2020). These scans are then merged into a single, three-dimensional geometrical mesh, which captures the shape and texture (i.e., the colors of skin, facial features, hair, and other detail) of their head. These avatars capture a person’s appearance with high accuracy, but their faces bear a neutral expression and are immobile. Our new construction pipeline builds on this process by providing these avatars with moving eyes and facial expressions. This pipeline is illustrated in Fig. 1 and summarized below, while a more detailed overview can be accessed in Appendix 1. For the purpose of explanation, this procedure can be broken into two broad stages -- the creation of avatar appearance (summarized in steps 1–5 below) and the rigging of the avatar’s face for eye gaze and facial expressions (steps 6–10). A manual detailing the full construction pipeline and a timelapse video of this procedure can also be accessed here: https://osf.io/xapwj/

**Fig. 2:**
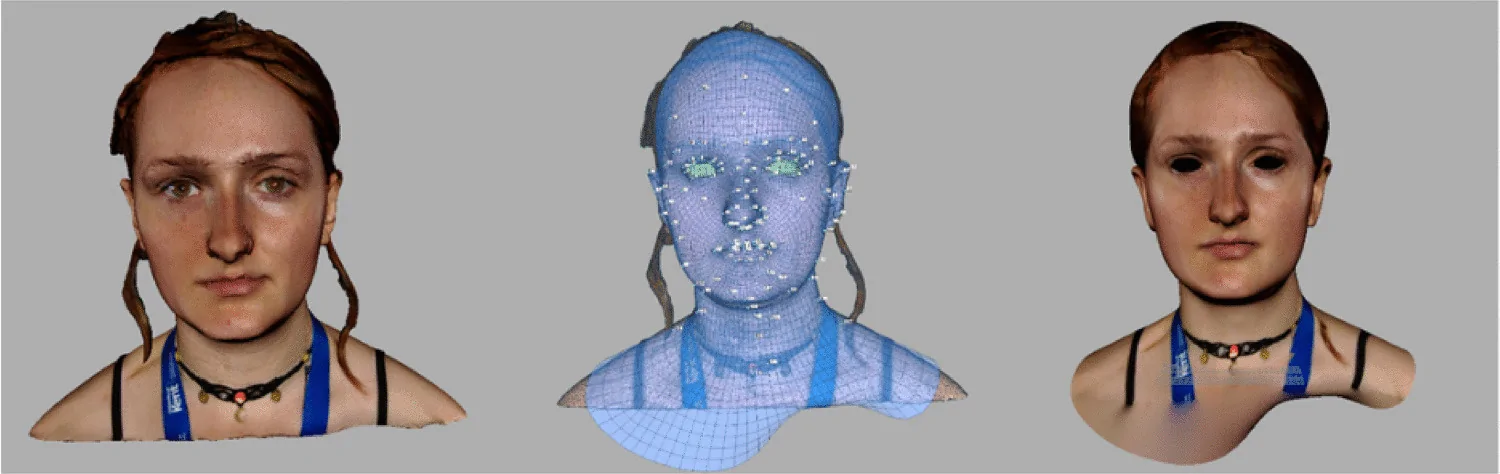
Overview of Step 1, showing how facial landmarks of the raw scan (***left***) are mapped to a template (***middle***) to create a wrapped scan with neater topology (***right***)

**Fig. 3:**
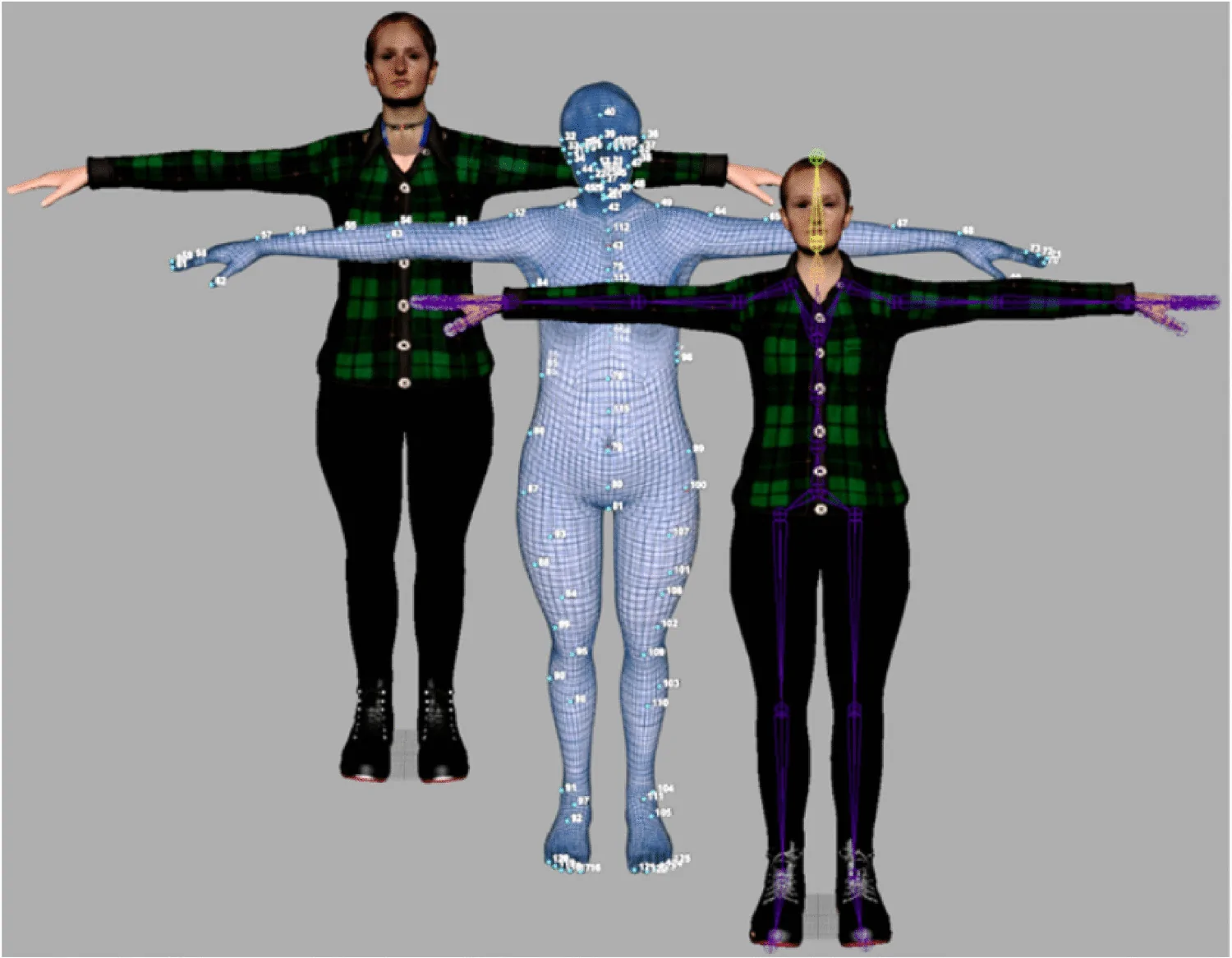
Overview of Steps 3 to 5, showing the process of combining the head and body and wrapping to create an avatar with corrected textures and a neater topology

**Fig. 4:**
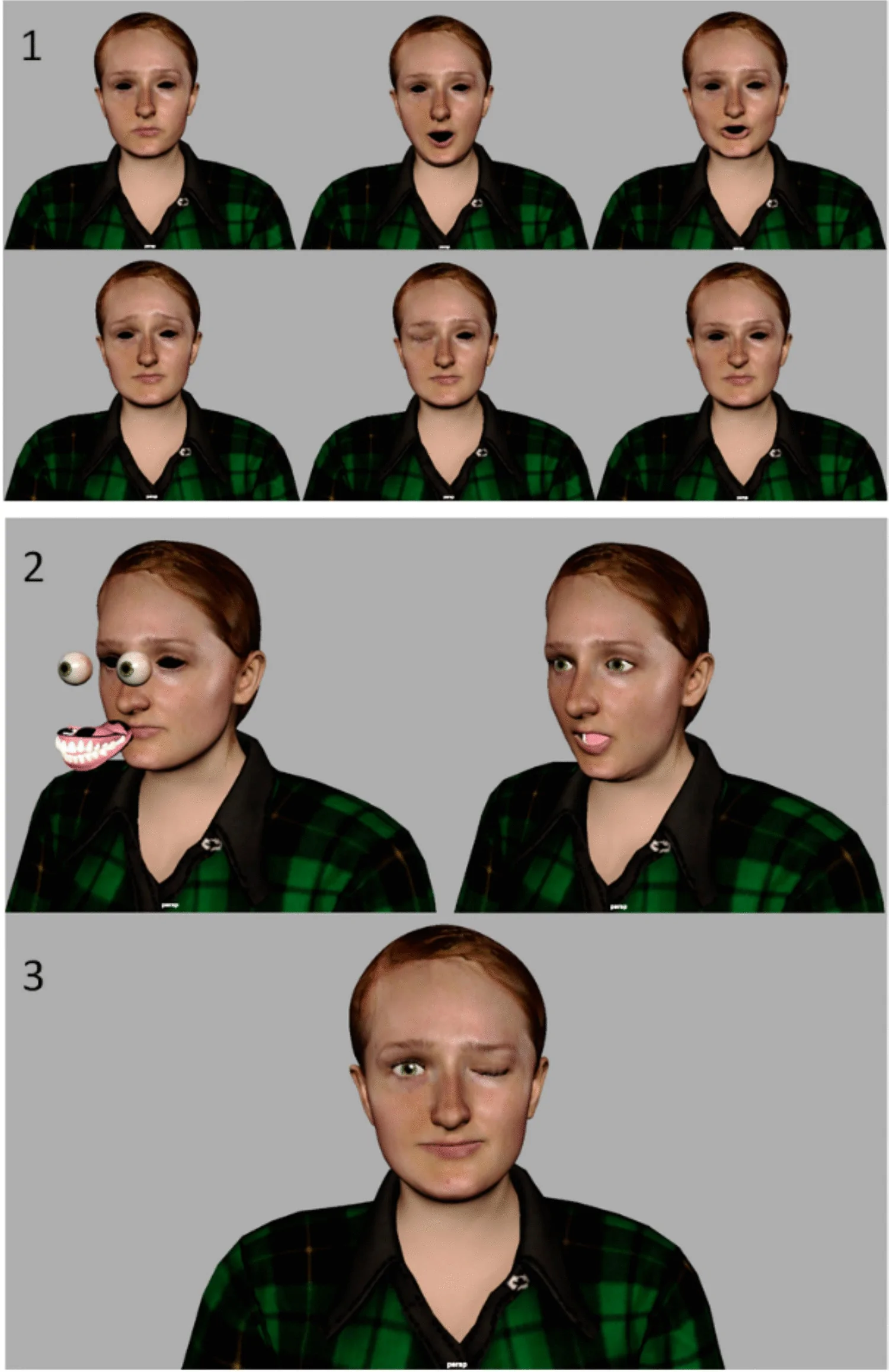
Overview of Steps 7 and 8, showing blend shape examples (**1**) and inserting eyes and teeth (**2**) to create an avatar with full facial rigging (**3**)

**Fig. 5:**
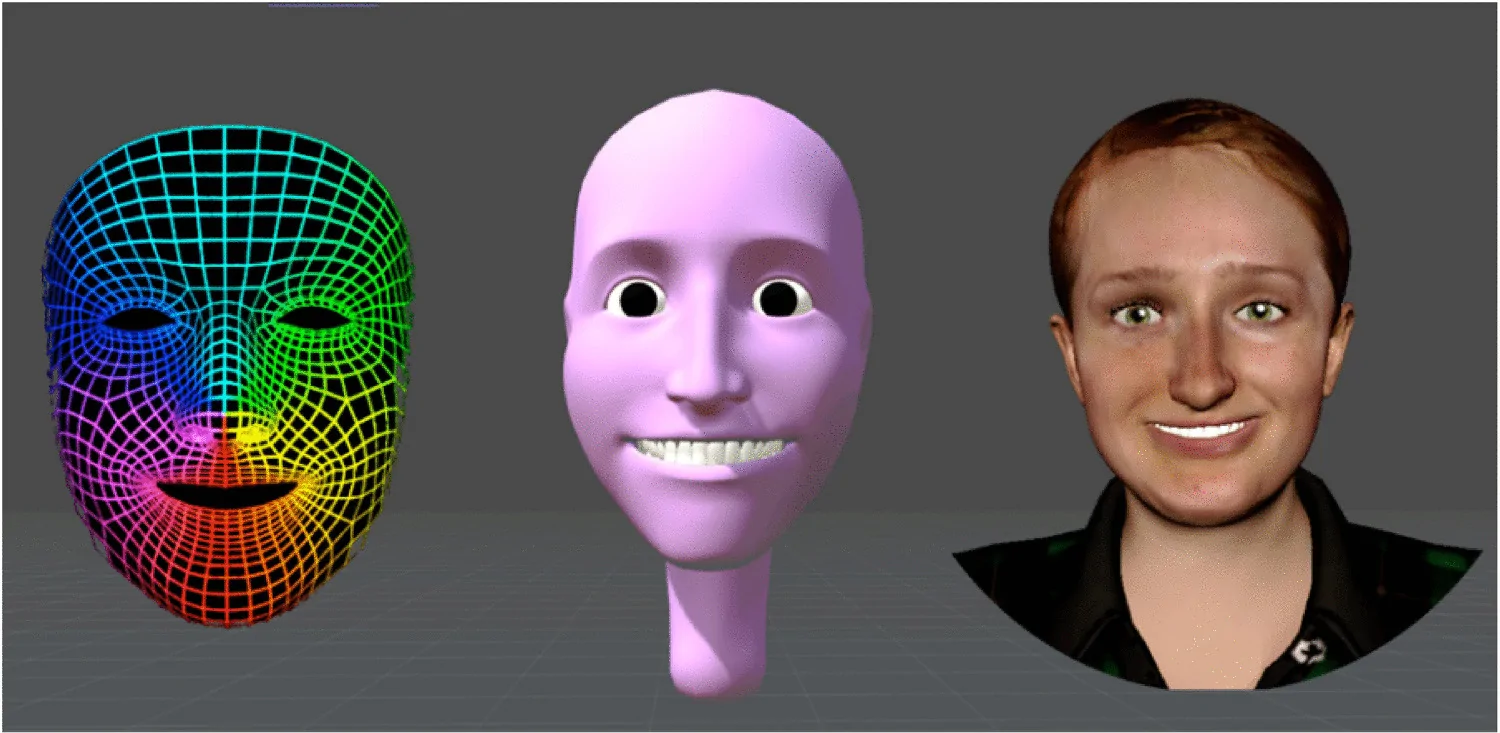
Illustration of Step 10, showing the recording of a real facial expression on the Rokoko iPhone app (***left***), its transfer onto a default model (***middle***), and the result on the avatar (***right***)

- **Step 1:** This step is designed to standardize the topology of avatar heads. This allows for the face mesh of avatars^1^ to be deformed more neatly, which is critical for animation of facial expressions. After acquisition of the 3D face scan of a person, the topology^2^ of this scan is standardized (using Faceform Wrap software; see Faceform LLC, 2023). During this process, landmarks such as the eyes, nose, and mouth are mapped between the initial face scan and a template face mesh. The template face mesh is then ‘wrapped’ onto the face scan to retain the geometry^3^ of the face scan while standardizing its topology. During this process, the eyes are also replaced with eye sockets and a mouth cavity is introduced to facilitate later animation (see Fig. 2). The wrapped face scan is exported, along with a new texture file (for full detail, see Chapters 1 and 2 of the manual accompanying this article).
- **Step 2**: In this step, an avatar body is then generated using a free character creator (Mixamo Fuse; see Mixamo Inc., 2013). This provides a variety of preset heads, arms, torsos, legs, and clothing, which can be selected and adjusted to personalize the avatar (for full detail, see Chapter 3 of the manual).
- **Step 3:** The wrapped face scan and avatar body are then combined into a single mesh and texture (using Autodesk Maya; see Autodesk Inc., 2022). For this purpose, the head of the Mixamo Fuse avatar and excess mesh from the shoulders of the wrapped face scan are removed to avoid overlap (Chapter 4).
- **Step 4:** This step ensures that male and female avatars have a uniform topology with other male- and female-presenting avatars. The standardized topology is important for applying blend shapes^4^ to the avatar later. For this purpose, the combined avatar mesh and textures are mapped between a template mesh and the combined avatar mesh (using Faceform wrap). The template mesh is then ‘wrapped’ onto the avatar mesh, to retain the geometry of the avatar while applying the standard topology of the template (Chapter 5).
- **Step 5:** The aim of this step is to create a more seamless transition between the texture of the avatar head and its body. This is necessary as the avatar heads were created separately from the body, before they were combined in Steps 3 and 4. The separate texture files for the face and body are imported as two layers in image processing software (GIMP v2.10.36; GIMP Development Team, 2023). Textural artifacts from the scanning process, such as black spots or clothing, are removed. Color adjustments are made so that the avatar body texture and face texture match, and the seams between the head and neck are blended together to create a more seamless transition between the head and body (Chapter 6). An illustration of steps 3–5 can be seen in Fig. 3.
- **Step 6:** The avatar is imported into auto-rigging software (Mixamo.com) so that it can be equipped with a standard skeletal rig and animations (e.g., sitting, idling) to create body movements (Chapter 7).
- **Step 7:** The aim of this step is to apply a standardized set of blend shapes to the avatars’ faces. This enables the direct mapping of dynamic emotional expressions from real faces onto the avatars (see Spring, 2020, for an overview). First, 52 blend shapes are generated for a male and female face template mesh (using Avatary’s Autoface module v1.16.0; FaceGood, 2023). These blend shapes are then mapped from the template mesh onto the rigged avatar. By equipping avatars with these standardized blend shapes, it then becomes possible for dynamic facial expressions from a human face to be captured with a smartphone and mapped directly onto the avatar mesh (Chapter 8).
- **Step 8:** The purpose of this step is to insert eyes and teeth models into the avatar. Blend shapes are created for the eyes to reflect different looking directions (i.e., up, down, left, right). A blend shape for the teeth is also made to reflect opening and closing of the jaw. The movement of the eyes and teeth is then connected to the movement of the face blend shapes. This enables movement captured from recordings of facial expressions to be mapped onto the eyes and teeth as well as the face itself, ensuring anatomically consistent facial movement (Chapter 9). Steps 7 and 8 are illustrated in Fig. 4.
- **Step 9:** In this step, eyelashes are created, placed, and shaped on the eyelids of the avatar (using Autodesk Maya’s XGen module). Then, eyelashes are rigged to follow the movement of the eyelids. After rigging, the eyelash meshes are duplicated to create various blend shapes that reflect different movements of the eyelids, such as looking down, blinking, or widening of the eyes (Chapters 10 and 11).
- **Step 10:** Finally, to provide the avatars with facial movement, real emotional expressions of a person are recorded on a mobile device (using the Rokoko Face Capture application and software; see Rokoko Electronics ApS, 2024). The recorded expressions are then transferred directly onto the avatar mesh (see Fig. 5). Fine adjustments can be made to the weighting of different blend shapes (using the Maya plugin Animbot version 2.4.8; Camilo., 2024) to reduce artifacts and make the appearance of expressions natural in appearance (Chapters 12 and 13).

## Experiment 1

To validate the facial animations of our avatars, we conducted two experiments assessing the perception of gaze and facial expressions. In Experiment 1, we examined whether participants orient their attention in response to avatar gaze cues. Human eyes naturally guide observers’ visual attention - a phenomenon known as the *gaze-cueing effect* (Driver et al., 1999; Friesen & Kingstone, 1998). Gaze cueing is a robust characteristic of face perception and human behavior (for a review and meta-analysis, see McKay et al., 2021). Here, we tested whether avatars constructed with our method elicit comparable attention shifts.

For this purpose, we employed two avatars that were created using our construction pipeline. We presented these avatars, one at a time, within an immersive virtual environment. On each trial, an avatar averted its gaze leftward or rightward, followed by the appearance of a target probe on either the cued (congruent) or opposite (incongruent) side. This technique examines whether participants show faster responses to congruent targets, replicating classic gaze-following effects observed with real faces (Bayliss et al., 2004; Driver et al., 1999; Friesen & Kingstone, 1998; Lachat et al., 2012). The effects are often observed with very short stimulus-onset asynchronies (SOAs) between a directional shift in eye-gaze and the appearance of a target (Frischen et al., 2007; Gregory & Jackson, 2021), and so the question arises whether avatars can also elicit such fast gaze shifts. Finally, we also included questionnaires to measure social presence, to explore the extent to which the avatars were perceived as human-like (Bailenson et al., 2003; Lombard et al., 2009), and participants’ sense of immersion in the virtual environment (O’Brien et al., 2018).

### Method

#### Participants

A meta-analysis of gaze-cueing effects shows that localization tasks produce small to moderate average effect sizes (Hedges’ *g* = .34, see McKay et al., 2021). The sample size was therefore determined a priori using G*Power for a 2 (gaze congruency) × 3 (SOA) within-subjects ANOVA with an alpha level of .05 and a power of .95, and a small to moderate effect size of .175. This revealed a minimum required sample size of *N* = 55. Accordingly, 55 participants (32 female, 23 male) aged between 18 and 65 years old (*M* = 24.9*, SD* = 7.85) took part in the study. Six were undergraduate psychology students recruited through the University of Kent Research Participation Scheme and participated in exchange for course credits. In addition, 49 paid participants were recruited through the university’s job advertisement platform and word-of-mouth. The study was approved by the University of Kent School of Psychology Ethics Committee and pre-registered on the Open Science Framework (https://osf.io/jn3ky/). All participants gave informed consent before participating and were free to withdraw their consent at any point without consequences. All participants reported normal or corrected-to-normal vision.

#### Stimuli

**Fig. 6:**
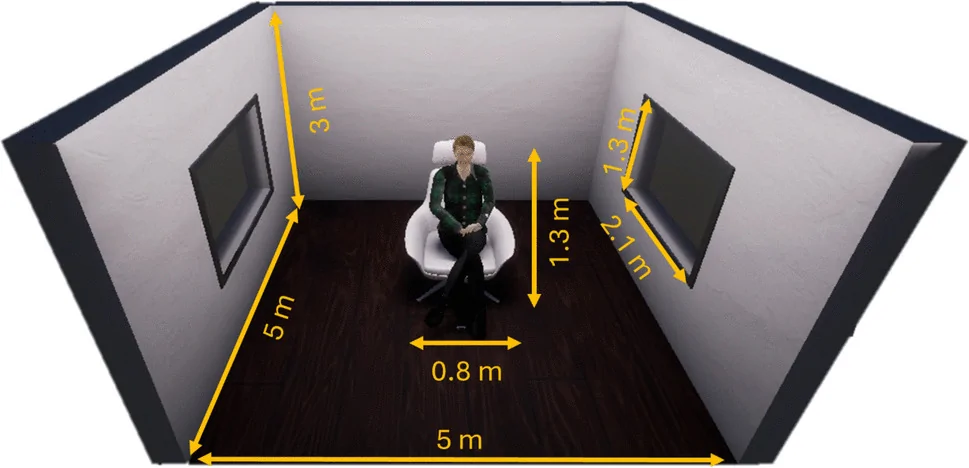
Overview of the virtual room. *Note.* One unit in Unity is roughly equal to one real-world meter (Unity Technologies, 2018)

**Fig. 7:**
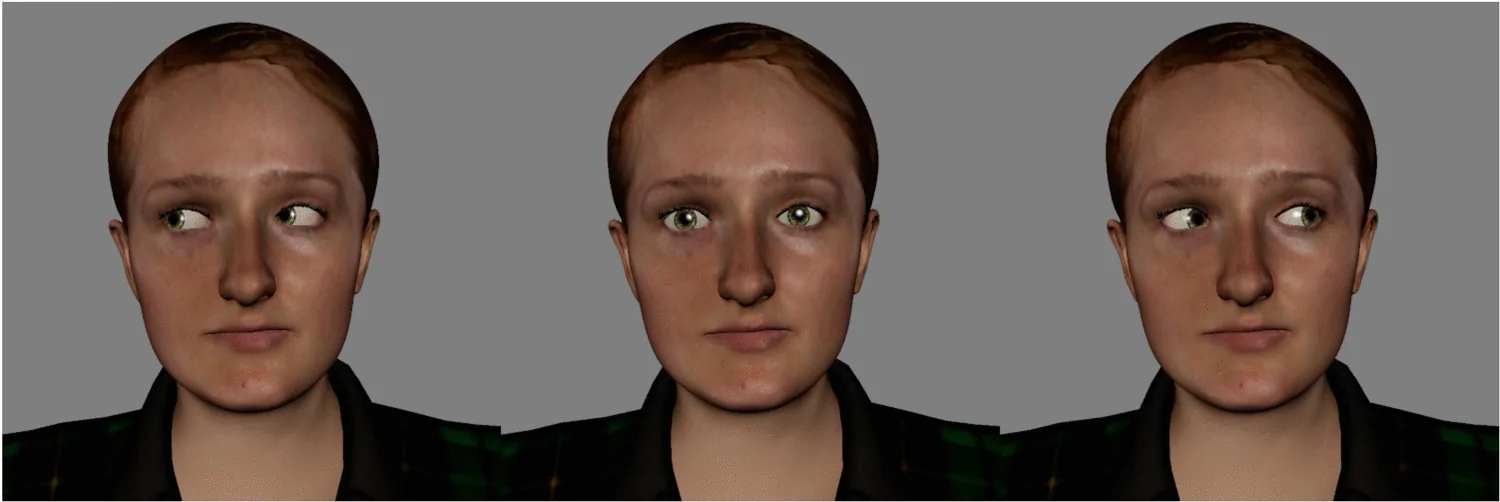
Example of an avatar with direct and averted eye-gaze

One male and one female avatar were generated using the high-fidelity 3D scanning pipeline described earlier. The target stimuli were grey spheres that appeared 30 cm to the left or right of the avatar’s face (*x*-axis), at a height of 1.1 m from the floor (*y*-axis), and 0.2 m in front of the avatar (*z*-axis). In the experiment, these avatars were viewed sitting on a chair in the center of a minimalist virtual room (see Fig. 6 for an illustration of the room and its key dimensions). The virtual camera, representing the participant’s viewpoint, was positioned ~ 0.7 m from the avatar at eye level (1.4 m). Slight variations occurred across participants due to individual headset adjustments. An illustration of an avatar’s gaze patterns can be seen in Fig. 7.

#### Procedure

The experiment was run using Unity 2022.3.28f1 in a 2 (congruence: congruent, incongruent) × 3 (SOA: 100, 300, 700 ms) within-subject design. Participants wore a Meta Quest 2 headset to complete the task in virtual reality. The experimenter initiated each trial with a keypress, which triggered an avatar in a chair in the center of the room to avert its gaze (direct gaze) to the right or to the left (averted gaze). After a variable delay depending on the cue-target SOA for that trial (100, 300, or 700 ms), the target appeared for 500 ms and was either in line with the avatar’s gaze (congruent trials, see Fig. 7) or on the opposite side (incongruent trials). Participants were asked to indicate whether the target spheres appeared on the left or right side using corresponding buttons on handheld controllers. All participants completed 24 practice trials and 120 experimental trials (20 trials for each combination of congruence and SOA) per avatar. For practice trials only, participants received audio feedback on the accuracy of their answers to ensure they understood the task and the controls. Once a response had been registered, the avatar’s gaze reset to the center until the next trial was initiated by the experimenter. Within the practice and the experimental block of trials, the order of trials was randomized and distributed evenly across avatars. Gaze direction was non-predictive of target location. The order in which participants saw the avatars was counterbalanced between participants.

#### Questionnaires

Following the gaze-cueing task, participants completed measures of social presence and immersion. Social presence was assessed using Bailenson et al.’s (2003) six-item scale as well as the Social Presence subscales (Parasocial, Passive and Active subscales; 14 items) of the Temple Presence Inventory (TPI, Lombard et al., 2009). Both Social Presence measures record participants’ responses on seven-point Likert scales. For Bailenson et al.’s (2003) measure, the scale ranged from - 3 “Strongly Disagree” to + 3 “Strongly Agree” (e.g., “I perceive that I am in the presence of another person in the room with me.”). For the Temple Presence Inventory, the scale ranged from 1 to 7, with varying response scale anchors depending on whether the item regarded level of agreement or frequency (e.g., “How often did you want to or did you make eye-contact with someone you saw?”, 1 being “Never”, 7 being “Always”).

In addition to social presence, immersion was measured using the refined User Engagement Scale Long-Form (rUES-LF, O’Brien et al., 2018). This scale consists of 30 statements with which participants indicated their level of agreement on a five-point scale ranging from 1, “Strongly Disagree” to 5 “Strongly Agree”, with 3 being “Neither Agree nor Disagree”. The scale consists of four components evaluating focus of attention (seven items, e.g., “*I lost myself in this experience*”), perceived usability (eight items, e.g., “*I felt frustrated while in the virtual room*”), aesthetic appeal (five items, e.g. “*This virtual room was attractive*”) and reward factor (ten items, e.g., “*Using this application was worthwhile.*”). The full questionnaires can be viewed in Appendix 2.

### Results

#### Gaze cueing

**Fig. 8:**
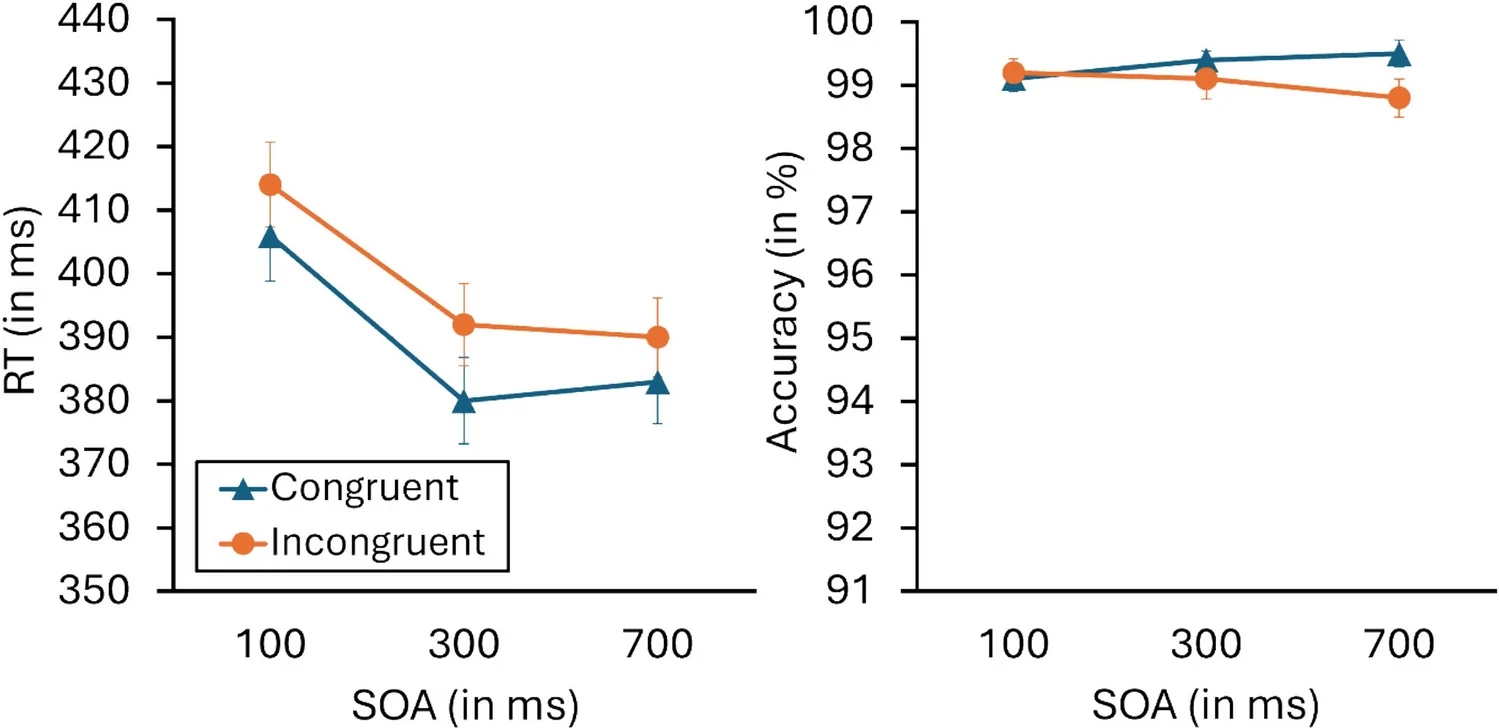
Response times and accuracy for the congruent and incongruent eye-gaze conditions as a function of SOA in Experiment 1. *Note. Error bars* denote the standard error of the means

The primary measure of interest was participants’ median response times to the target spheres as a function of eye-gaze congruency and stimulus-onset asynchrony (SOA). The cross-subjects means of these data are provided in Fig. 8. A 2 (congruency: congruent, incongruent) × 3 (SOA: 100, 300, 700 ms) within-subjects ANOVA of these data revealed a main effect of congruency, *F*(1, 54) = 42.35,* p* < .001, ${\eta}_{p}^{2}=.44$, due to faster RTs on congruent (*M* = 390 ms, *SD* = 46) than incongruent eye-gaze trials (*M* = 399 ms, *SD* = 50). A main effect of SOA was also observed, *F*(2, 108) = 82.40,* p* < .001, ${\eta}_{p}^{2}=.60$*.* Bonferroni *t* test indicated that responses were faster with a 300 ms SOA (*M* = 386 ms, *SD* = 48) than a 100 ms SOA (*M* = 410 ms, *SD* = 51), *t*(54) = 14.20, *p* < .001, *d* = 0.49, and with a 700 ms SOA (*M* = 386 ms, *SD* = 47) than a 100 ms SOA, *t*(54) = 9.48, *p* < .001, *d* = 0.48. In contrast, RTs were comparable for the 300 ms and 700 ms SOAs, *t*(54) = 0.19,* p* = 1.00, *d* = 0.01. The interaction of congruency and SOA was not significant, *F*(2, 108) = 1.62, *p* = .202, ${\eta}_{p}^{2}=.03$.

To compare accuracy in the gaze-cueing task across conditions, the percentage of correct responses was also calculated for every participant for each condition. The cross-subject means of these data can be seen in Fig. 8 and show that accuracy was uniformly high. Accordingly, a 2 (congruency) × 3 (SOA) within-subject ANOVA showed no main effect of congruency, *F*(1, 54) = 1.73, *p* = .194, ${\eta}_{p}^{2}=.03$, no main effect of SOA, *F*(1, 54) = 0.25, *p* = .776, ${\eta}_{p}^{2}=.01$, and no interaction between these factors, *F*(2, 108) = 1.96, *p* = .146, ${\eta}_{p}^{2}=.04$.

#### Social presence

We then explored whether perceived social presence of avatars moderated gaze cueing. For this purpose, responses to the Bailenson et al. (2003) social presence measure were transposed from the numerical scale ranging from – 3 to + 3 to the same 1-to-7 scale as the Temple Presence Inventory, and the mean of participants’ scores across both questionnaires was calculated. The items of the Temple Presence Inventory addressing sound (Items 3, 5, and 9) were excluded from this analysis as auditory information was not provided in the experiment. Based on these scores, the sample of participants was then divided into three groups, which reflected those who reported low (*N* = 19, *M* = 2.64, *SD* = 0.36), medium (*N* = 15, *M* = 3.46, *SD* = 0.17) or high (*N* = 21, *M* = 4.49, *SD* = 0.79) social presence of the avatars. In this analysis, participants who reported a social presence inferior to the 33rd percentile (M = 3.12) or superior to the 67th percentile (M = 3.76) were assigned to the low and high social presence groups, respectively, while the remaining participants were assigned to the medium group.

**Fig. 9:**
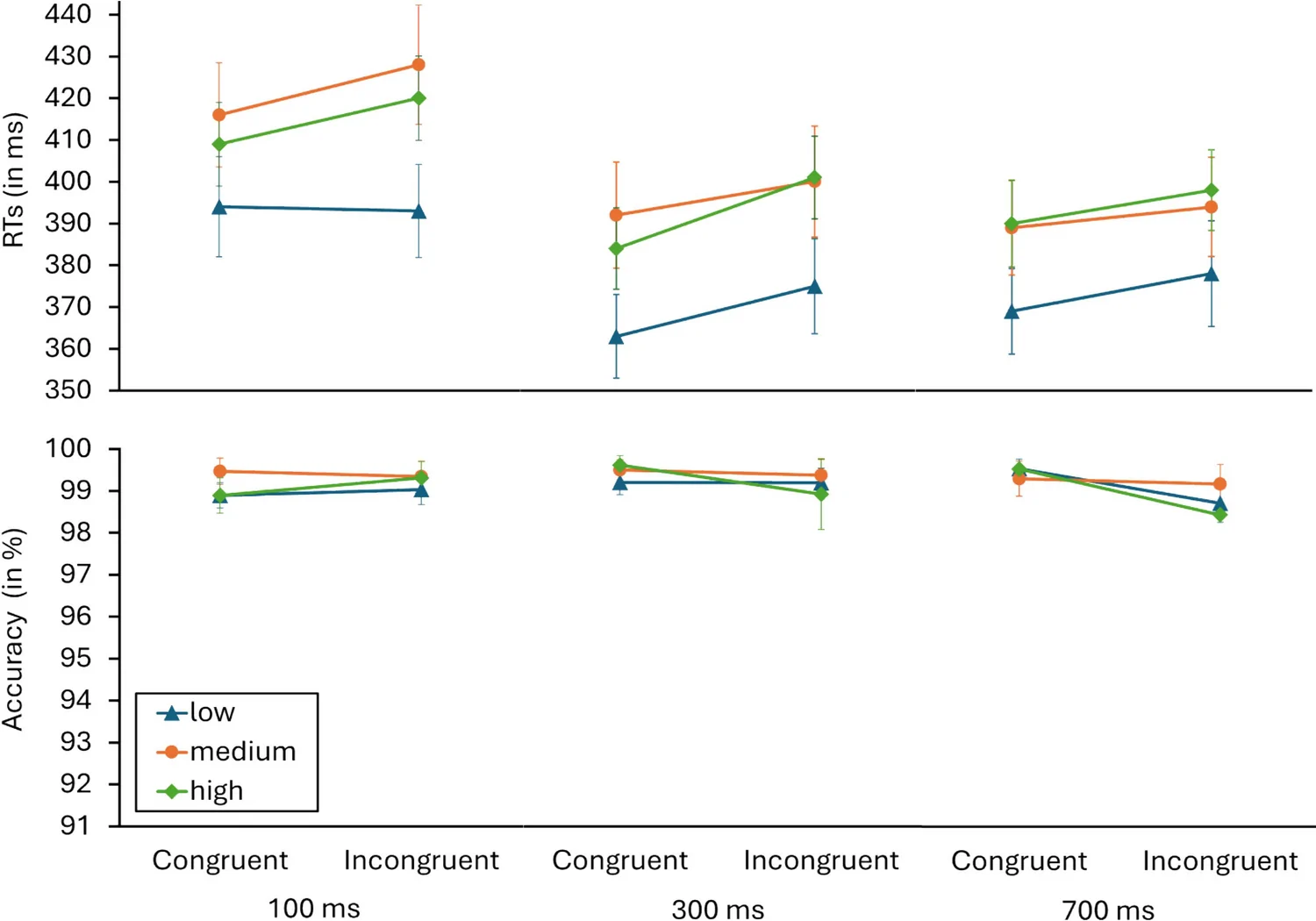
Response times (***top***) and accuracy (**bottom**) for the congruent and incongruent eye-gaze conditions as a function of social presence and SOA in Experiment 1. *Note. Error bars* denote the standard error of the means

The speed and accuracy of target localization were then calculated for these three groups (see Fig. 9) and 2 (congruency: congruent, incongruent) × 3 (SOA: 100, 300, 700 ms) × 3 (SP score: low, medium, high) mixed-factor ANOVAs were conducted on response times and accuracy. For response times, this analysis again revealed a main effect of congruency, *F*(1, 52) = 43.06,* p* < .001, ${\eta}_{p}^{2}=.45$, and a main effect of SOA, *F*(2, 104) = 84.45,* p* < .001, ${\eta}_{p}^{2}=.62$, but no main effect of social presence, *F*(2, 52) = 1.83,* p* = .170, ${\eta}_{p}^{2}=.07$. None of the interactions between these factors reached significance, all *Fs* ≤ 2.75, all *ps* ≥ .073, *all* ${\eta}_{p}^{2}\le .10$.^5^ A corresponding 2 (congruency) × 3 (SOA) × 3 (social presence) mixed-factor ANOVA for accuracy showed no main effects of congruency, *F*(1, 52) = 1.55, *p* = .219, ${\eta}_{p}^{2}=.03$, SOA, *F*(2, 104) = 0.29, *p* = .747, ${\eta}_{p}^{2}=.01$*,* or social presence, *F*(2, 52) = 0.07, *p* = .935, ${\eta}_{p}^{2}=.00$, and no interactions between these factors, all *Fs* ≤ 1.63, all *p*s ≥ .202, *all* ${\eta}_{p}^{2}\le .03$.

#### Immersion

**Fig. 10:**
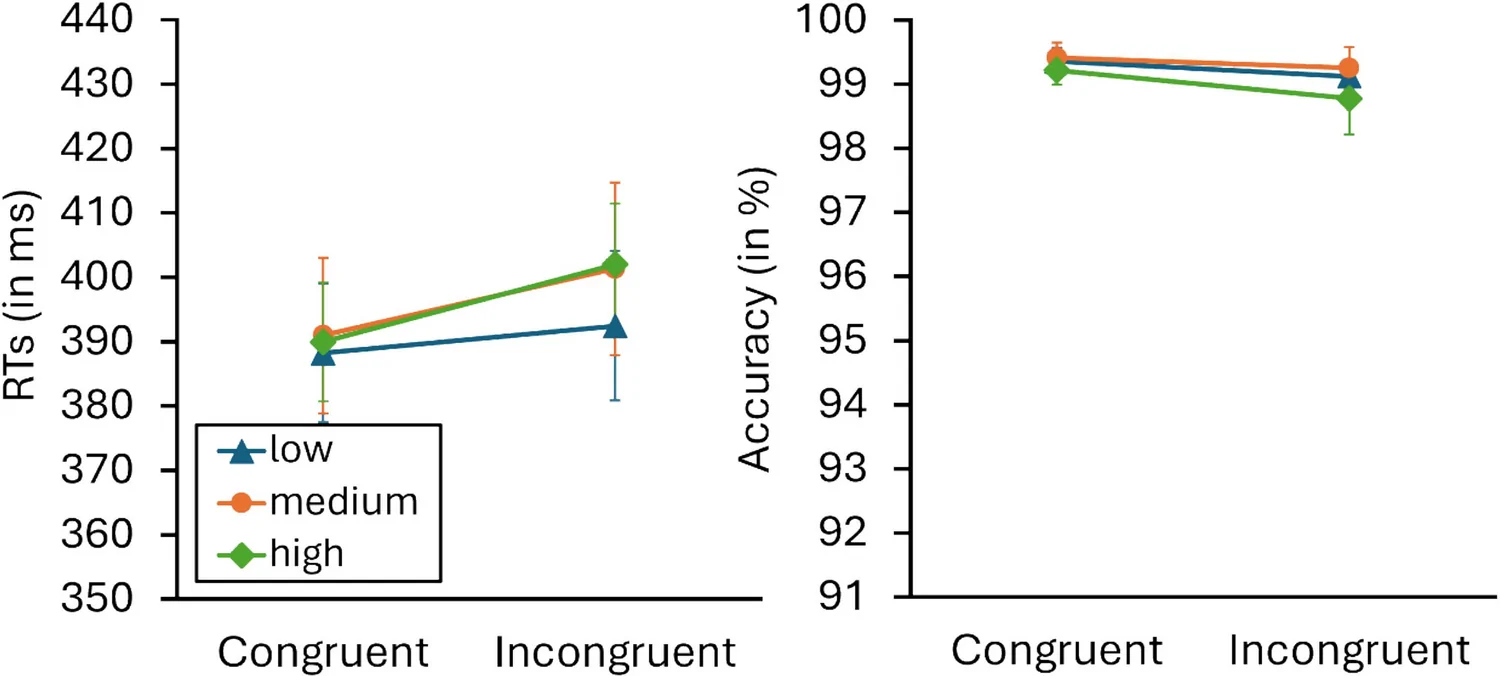
Response times and accuracy for the congruent and incongruent eye-gaze as a function of immersion in Experiment 1. *Note. Error bars* denote the standard error of the means

As an additional step of the exploratory analysis, we also investigated whether immersion influenced gaze cueing. For this purpose, an immersion score was calculated from the mean of participants’ individual responses on the refined User Engagement Scale Long-Form. Based on these scores, the sample of participants was then divided into three groups, which reflected those who reported low (*N* =18, *M* = 2.22, *SD* = 0.21), intermediate (*N* = 20, *M* = 2.81, *SD* = 0.13), or high (*N* =17, *M* = 3.42, *SD* = 0.28) immersion in the environment. In this analysis, participants who reported an immersion inferior to the 33rd (*M* = 2.55) or superior to the 67th (*M* = 3.00) percentile were assigned to the low and high levels, respectively, while the remaining participants were assigned to the medium group. The speed and accuracy of target localization were then calculated for these three groups (see Fig. 10). Two 2 (congruency: congruent, incongruent) × 3 (SOA: 100, 300, 700 ms) × 3 (immersion: low, medium, high) mixed-factor ANOVAs were then conducted on accuracy and on RTs.

For response times, ANOVA revealed a marginal interaction between congruency and immersion, *F*(2, 52) = 3.18, *p* = .050, ${\eta}_{p}^{2}=.11$. Bonferroni *t* test showed faster response times to targets congruent than incongruent with avatar gaze when participants experienced intermediate, *t*(52) = 4.75, *p* < .001, *d* = 0.21, or high immersion,* t*(52) = 5.12, *p* < .001, *d* = 0.24. In contrast, participants who reported lower immersion did not exhibit a gaze-cueing effect, with similar response times for congruent and incongruent gaze-target locations, *t*(52) = 1.85, *p* = 1.00, *d* = 0.09. No main effect of immersion, *F*(2, 52) = 0.08, *p* = .919, ${\eta}_{p}^{2}=.00$, or further interactions were found, all *F*s ≤ 1.56, *p*s ≥ .214, all ${\eta}_{p}^{2}\le .03$.

For accuracy, a 2 (congruency) × 3 (SOA) × 3 (immersion) mixed-factor ANOVA showed no main effects of congruency, *F*(1, 52) = 1.76, *p* = .191, ${\eta}_{p}^{2}=.03$, SOA, *F*(2, 104) = 0.20, *p* = .816, ${\eta}_{p}^{2}=.00$, or immersion, *F*(2, 52) = 0.45, *p* = .639, ${\eta}_{p}^{2}=.02$, and no interaction between these factors, all *F*s ≤ 2.06, all *p*s ≥ .132, all ${\eta}_{p}^{2}\le 0.07$.

### Discussion

Experiment 1 evaluated whether avatars constructed with our pipeline elicit gaze-cueing effects comparable to those observed with real human faces. Consistent with prior findings (e.g., Driver et al., 1999; Friesen & Kingstone, 1998; McKay et al., 2021), participants were faster to detect targets appearing in the direction of avatar gaze than in the opposite direction. This effect emerged even at an SOA of 100 ms, indicating rapid and potentially reflexive orienting to avatar gaze cues (see, e.g., Driver et al., 1999; Friesen & Kingstone, 1998). We also explored whether the magnitude of gaze cueing was moderated by the perceived social presence of avatars and immersion in VR. Social presence did not demonstrate a reliable relationship with gaze cueing, which converges with evidence that gaze cueing can be elicited even by face stimuli of low realism and person presence (see, e.g., Friesen & Kingstone, 1998). In contrast, gaze cueing appeared to be dependent on participants’ sense of immersion, which broadly reflects the extent to which they felt physically present within the virtual environment. Together, these results indicate that the eye-gaze behavior of avatars created using our method is readily perceived and acted upon by observers in ways closely paralleling responses to real human gaze.

## Experiment 2

In a second experiment, we examined whether avatars convey emotional facial expressions in ways comparable to the human faces from which they are derived. Emotion recognition is a crucial part of human social interaction (Izard, 1994; Riddell et al., 2024). Research in this domain typically uses six ‘basic’ emotions of happiness, sadness, fear, surprise, disgust, and anger (Ekman, 1972; Izard, 1971; Elfenbein & Ambady, 2002). However, recognition accuracy varies systematically between these emotions. Happiness and sadness, for example, are generally identified with high accuracy, whereas fear and disgust are often recognized less reliably (Elfenbein & Ambady, 2002).

In this experiment, we investigated whether avatars equipped with dynamic facial expressions using our modeling method produce emotion signals that can be accurately decoded by observers. For this purpose, participants classified the emotional expressions of avatars presented in immersive virtual reality. To provide benchmarks for comparison, participants also completed two emotion recognition tasks with facial photographs. One set consisted of images of the same individuals that were used to generate the avatars, thereby allowing a direct comparison between dynamic virtual and static photographic expressions. The other was the Warsaw Facial Expression Set (WFE; Olszanowski et al., 2015), a validated measure of emotion recognition based on photographs of the six basic emotions.

### Method

#### Participants

The sample size was determined a priori using G*Power for 3 (test: avatar, University of Kent Faces, Warsaw Facial Expressions) × 6 (facial expression: happy, sad, angry, fearful, surprised, disgusted) within-subjects ANOVA with an alpha level of .05 and a power of .95, and a medium effect size of f = .25. This revealed a minimum required sample size of* N* = 14, but as this is a small sample compared to previous studies we doubled this to 28 (e.g., *N* = 20 in Calder et al., 2001; *N* = 36 in Calder et al., 1996; *N* = 22 and *N* = 28 in Chamberland et al., 2017; *N* ≥ 29 in Ekman et al., 1987). Accordingly, 29 participants were recruited. However, due to a recording error, one participant was excluded from all analyses. The sample therefore comprised of 28 participants (23 female, four male, one preferred not to say) aged between 18 and 54 years (*M* = 20.1, *SD* = 6.7). Participants were undergraduate psychology students recruited through the University of Kent Research Participation Scheme and participated in exchange for course credits. The study was approved by the University of Kent School of Psychology Ethics Committee and pre-registered on the Open Science Framework (https://osf.io/yjfzx/). All participants gave informed consent before participating and were free to withdraw their consent at any point without consequences. All participants reported normal or corrected-to-normal vision.

#### Avatar expression test

**Fig. 11:**
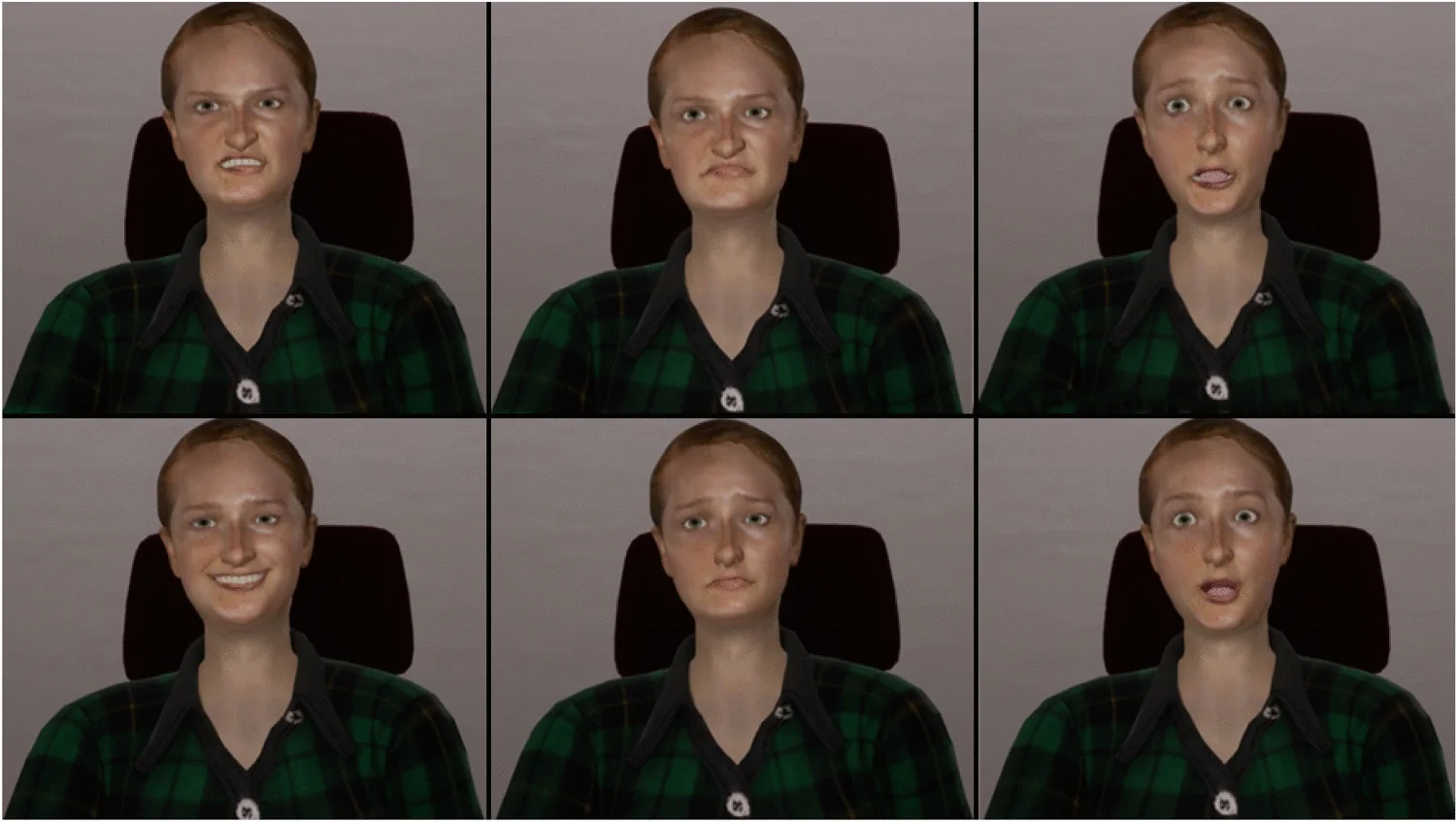
Example of an avatar’s facial expressions (from ***left to right*, *top row***: angry, disgusted, fearful; ***bottom row***: happy, sad, surprised

Four avatars (two female, two male) were created from high-fidelity 3D scans of real faces, following the construction method described in this article. Each avatar was rigged with six dynamic facial expressions corresponding to anger, fear, disgust, happiness, sadness, and surprise (see Fig. 11). These avatars were presented in four blocks, grouped by identity and counterbalanced across participants.

The experiment was run in Unity 2023.1.13f1 and displayed via a Meta Quest 2 headset. Each trial began with the avatar in a neutral state. The experimenter triggered the onset of one of the six emotional expressions, which unfolded dynamically across 2833 ms. During this time interval, each expression took 833 ms to reach peak intensity, maintained peak intensity for 1500 ms, and then returned to a neutral expression across 500 ms. Between trials, avatars displayed a neutral expression and also made naturalistic eye blinks every 3 s for added realism.

After each expression, participants verbally classified the emotion from the six alternatives. The experimenter recorded responses via a keypress and then initiated the next trial. Each of the four identities was presented in a separate block of trials, with order counterbalanced between participants. Within each of these blocks, each expression appeared ten times per identity, yielding a total of 240 trials (60 per identity, 40 per emotion). Immediately after this task, participants completed the social presence questionnaires as in Experiment 1, comprising Bailenson et al.’s (2003) six-item questionnaire as well as the Social Presence subscales (Parasocial, Passive and Active subscales; 14 items) of the Temple Presence Inventory (TPI, Lombard et al., 2009).

#### Photograph expression tests

**Fig. 12:**
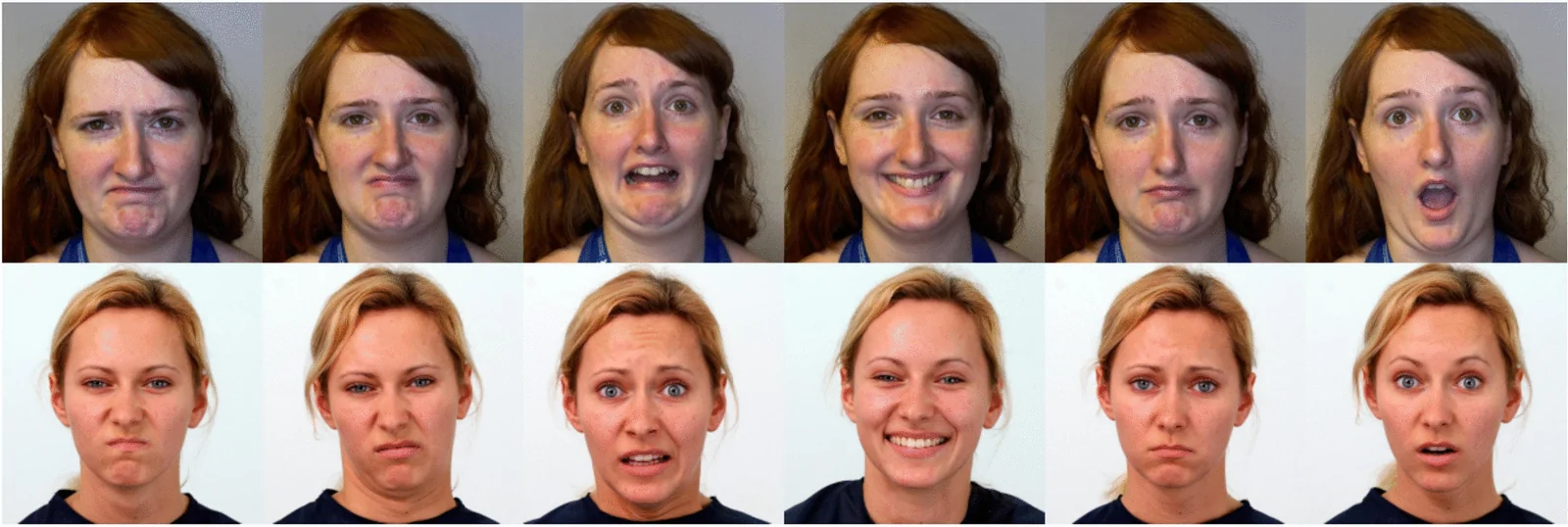
Examples of facial expressions from the University of Kent Faces (***top row***) and the Warsaw Facial Expressions (***bottom row***). From left to right: angry, disgusted, fearful, happy, sad, surprised)

To compare avatar expressions with real facial stimuli, two additional expression recognition tasks were administered. To provide a comparison for the test of avatar facial expressions, face photographs were recorded of the same individuals posing the six emotions of anger, disgust, fear, happiness, sadness, and surprise. This resulted in a stimulus set of 24 photographs, comprising six expressions for each of the four identities. These stimuli are referred to here as the University of Kent (UKF) faces. All photographs were sized to 800 × 542 pixels at a resolution of 95 ppi. Example stimuli can be seen in Fig. 12.

In the experiment, these stimuli were displayed in the center of a computer screen on a grey background using PsychoPy software. Each trial began with a fixation cross for 1000 ms, followed by a stimulus, which remained onscreen until a response was registered. Participants were asked to classify these faces according to their expression, using six keys on a computer keyboard. In this manner, participants completed four blocks of 60 trials. Each identity was presented in a separate block, with order counterbalanced between participants, and facial expressions appeared in a randomized order within blocks.

For further comparison, a second expression test was also included, comprising the Warsaw Facial Expression set (WFE, Olszanowski et al., 2015). We selected four identities (two female, two male) portraying the same set of six facial expressions (see Fig. 12). For consistency, all photographs were sized to 800 × 542 pixels at a resolution of 95 ppi. Similarly to the previous set of photographs, participants completed four blocks of 60 trials. Each identity was presented in a separate block, with order counterbalanced between participants, and facial expressions appeared in a randomized order within blocks.

### Results

#### Accuracy

To compare accuracy across tasks, the percentage of correct responses was calculated for all facial expressions. The cross-subjects means of these data can be seen in Table 1. Accuracy was generally high across tests and expressions. A series of one-sample *t* tests confirmed that participants performed above chance (i.e., 16.7%) for all six emotions in all tasks, all *t*s ≥ 13.54, all *p*s < .001, all *ds* ≥ 2.56.

**Table 1:** Mean (SD) accuracy per facial expression for each task

	Angry	Disgusted	Fearful	Happy	Sad	Surprised
VR	77.5 (19.1)	73.3 (22.1)	68.4 (17.1)	99.7 (0.8)	91.3 (12.3)	82.4 (8.8)
UKF	79.8 (15.9)	87.6 (9.8)	80.7 (19.2)	99.8 (0.7)	95.3 (5.7)	91.8 (7.1)
WFE	91.2 (8.2)	92.7 (6.2)	91.5 (10.1)	99.8 (0.7)	98.9 (2.5)	91.6 (9.8)

To compare the accuracy of expression classification across tasks, a 3 (test: avatars, UKF, WFE) × 6 (facial expression: angry, disgusted, fearful, happy, sad, surprised) within-subject ANOVA was conducted. This showed a main effect of task, *F*(2, 54) = 47.66, *p* < .001, ${\eta}_{p}^{2}=.64$, a main effect of facial expression, *F*(5, 135) = 31.06, *p* < .001, ${\eta}_{p}^{2}=.54$, and an interaction between these factors, *F*(10, 270) = 6.40, *p* < .001, ${\eta}_{p}^{2}=.19$. To analyze this interaction, post-hoc comparisons were performed using Bonferroni *t* tests. A full breakdown of these comparisons can be found in Appendix 3. The main results are summarized here and show that happy faces were recognized more accurately than any other facial expression in all tests, all *t*s ≥ 4.35, all *p*s ≤ .027, all *d*s ≥ 0.38, except for sad faces in the WFE and the avatar test conditions,* t*(27) = 2.18,* p* = 1.00, *d* = 0.08 and *t*(27) = 3.70,* p* = .150, *d* = 0.72, respectively. Similarly, sad faces were recognized with greater accuracy than fearful expressions across all tests, all *t*s ≥ 4.15, all *p*s ≤ .046, all *d*s ≥ 0.62. This suggests that the accuracy with which facial expressions were classified followed a similar pattern for avatar faces as in the photos and WFE tests.

This is also evident when these tests are compared directly, which shows accuracy was comparable for angry, happy, and sad faces across all tests, all *t*s ≤ 3.80, all *p*s ≥ .116, all *d*s ≤ 1.16. For disgusted, fearful, and surprised expressions, the pattern was more variable. Avatar expressions of surprise were classified with similar accuracy to the WFE, *t*(27) = 3.42, *p* = .308, *d* = 0.79, but with lower accuracy than photos of the same identities, *t*(27) = 4.43, *p* < .05, *d* = 0.81. In contrast, avatar expressions of disgust were classified with similar accuracy to photos of the same identities, *t*(27) = 3.36, *p* = .355, *d* = 1.22, but with lower accuracy than WFE faces, *t*(27) = 4.84, *p* <. 01, *d* = 1.65. In addition, avatar expressions of fear were classified with lower accuracy than face photos on both comparison tests, both *t*s ≥ 4.99, *p*s < .01, *d ≥* 1.05.

#### Confusions

**Fig. 13:**
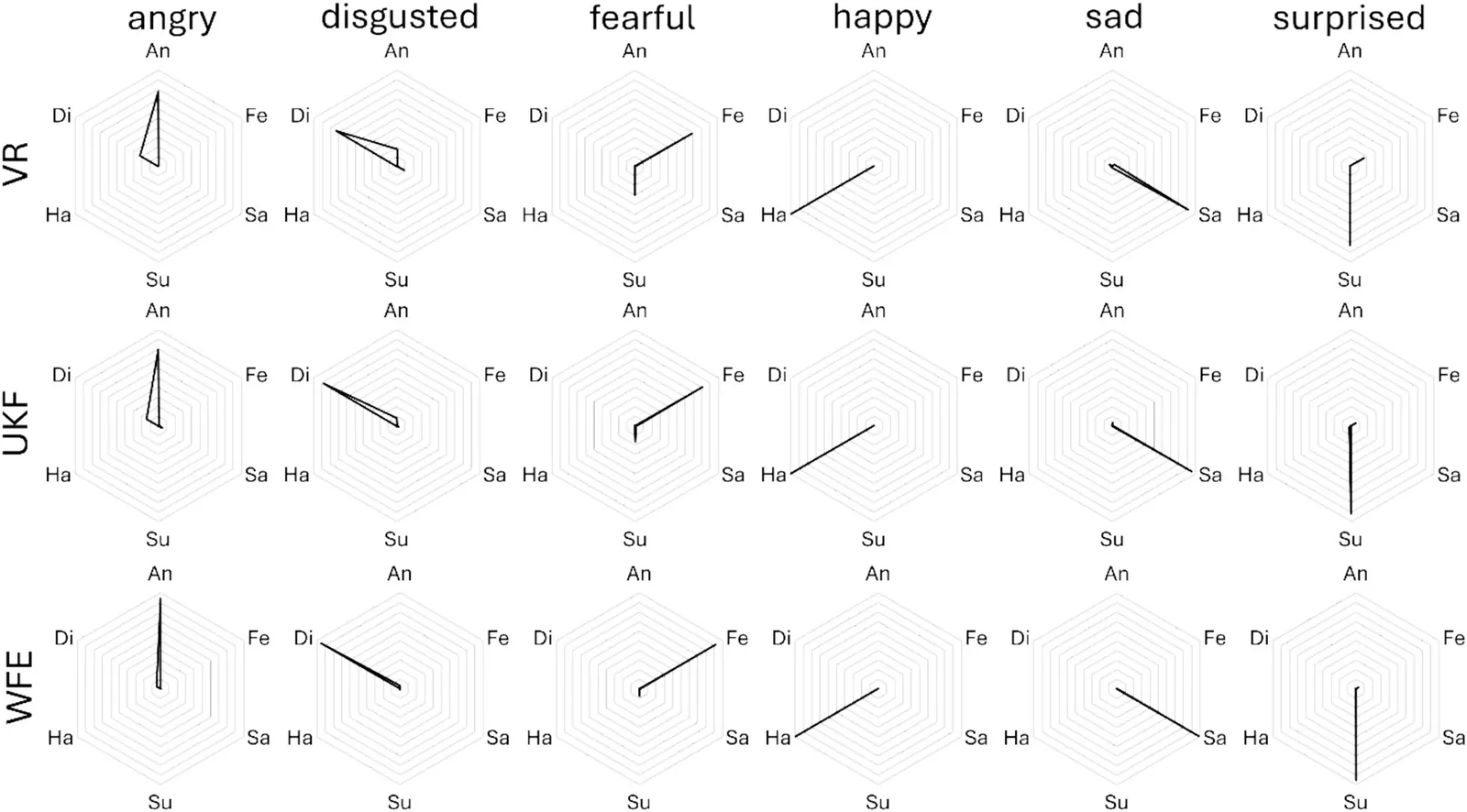
Response distribution per facial expression across tasks in Experiment 2. *Note.* An = angry, Di = disgust, Fe = fear, Ha = happy, Sa = sad, Su = surprise

To further explore whether facial expressions of avatars were perceived similarly to the face photos, the confusions between expressions were analyzed and are plotted in Fig. 13. This demonstrates a similar pattern of confusions across tests. For example, facial expressions of anger were most likely to be confused with disgust, and vice versa, across all three tests. Similarly, facial expressions of fear were most likely to be mistaken as surprise, and surprise as fear. Finally, in all three tests, accuracy was highest for facial expressions of happiness and sadness, and confusions of these expressions were low.

#### Correlations

**Fig. 14:**
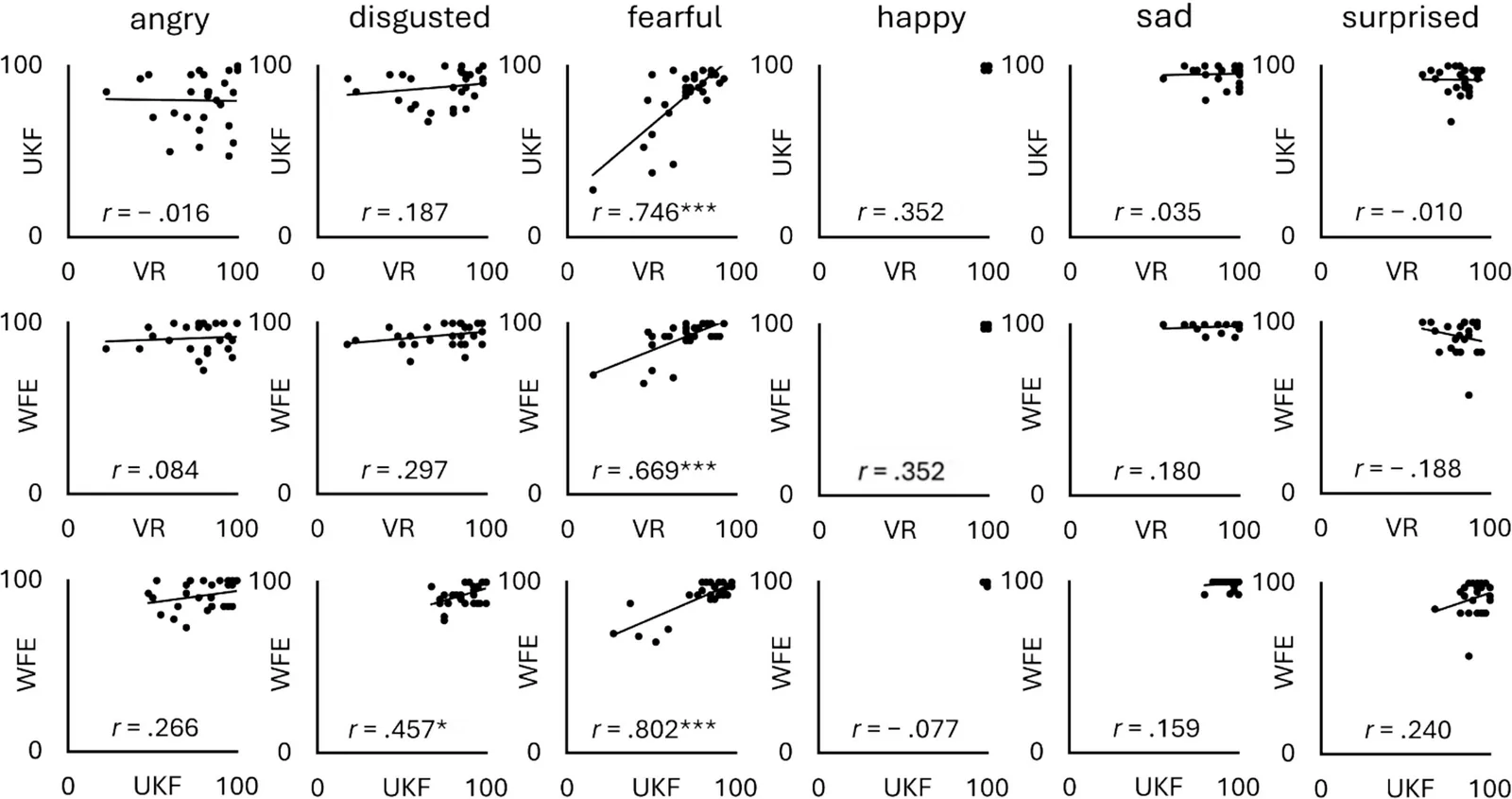
Response distribution per facial expression from 0 to 100% accuracy across tasks in Experiment 2. *Note.* * *p* < .05, ** *p* < .01, *** *p* < .001

To investigate the relationship between performance across tasks, a one-tailed correlation matrix between individual percentage accuracy in each task was analyzed. These correlations are illustrated in Fig. 14. As this data shows, only accuracy for fearful faces correlated across all tasks, all *p*s < .001. Performance between the WFE and UKF correlated for disgusted faces, *p* < .05. The other correlations were not significant, all *p*s > .05.

#### Social presence and immersion

**Fig. 15:**
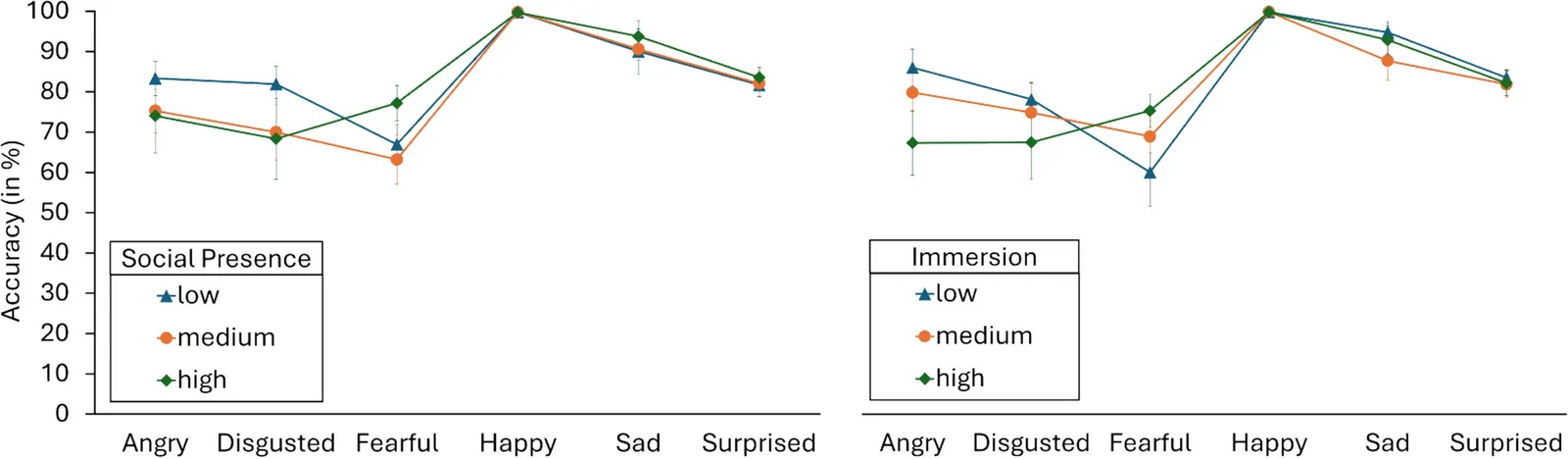
Accuracy scores per facial expression from 0 to 100% accuracy across social presence and immersion groups in Experiment 2

As an additional step, we again investigated whether the perceived social presence of avatars affected the accuracy with which participants recognized facial expressions. For this purpose, we again transposed the responses to Bailenson et al.'s (2003) social presence measure to a 1-to-7 scale, before calculating a composite score comprising the mean of participants’ scores on this questionnaire and the Temple Presence Inventory. Again, items relating to sound (Items 3, 5, and 9) were excluded as they were not applicable to this paradigm. Based on these scores, the sample of participants was then divided into three groups, which reflected those who reported low (*M* = 2.96, *SD* = 0.20), medium (*M* = 3.73, *SD* = 0.29), or high (*M* = 4.60, *SD* = 0.50) social presence of the avatars. For this analysis, participants who reported a social presence inferior to the 33rd (*M* = 3.29) or superior to the 67th percentile (M = 4.06) were assigned to the low and high social presence groups, respectively, while the remaining participants were assigned to the medium group. These data are shown in Fig. 15. A 3 (social presence: low, medium, high) × 6 (facial expression: angry, disgusted, fearful, happy, sad, surprised) mixed-factor ANOVA on the accuracy percentage for the avatar expressions revealed a main effect of expression, *F*(5, 125) = 17.00, *p* < .001, ${\eta}_{p}^{2}=.41$, but no main effect of social presence, *F*(2, 25) = 0.73, *p* = .494, ${\eta}_{p}^{2}=.06$, and no interaction between these factors, *F*(10, 125) = 0.94, *p* =. 504, ${\eta}_{p}^{2}=.07$.

Similarly, to investigate the role of immersion on accuracy, an immersion score was obtained by calculating participants’ mean scores across all scales of the UES-LF. Based on these scores, the sample of participants was then divided into three groups, which reflected those who reported low (*M* = 3.44, *SD* = 0.28), medium (*M* = 3.96, *SD* = 0.13), or high (*M* = 4.45, *SD* = 0.17) immersion in the environment (see Fig. 15). Participants who reported an immersion inferior to the 33rd (*M* = 3.80) or superior to the 67th percentile (*M* = 4.10) were assigned to the low and high immersion groups, respectively, while the remaining participants were assigned to the medium group. A corresponding 3 (immersion) × 6 (facial expression) mixed-factor ANOVA on the accuracy data in the virtual reality task revealed a main effect of expression, *F*(5, 125) = 18.61, *p* < .001, ${\eta}_{p}^{2}=.43$, but no main effect of immersion, *F*(2, 25) = 0.33, *p* = .725, ${\eta}_{p}^{2}=.03$, and no interaction between immersion and expression, *F*(10, 125) = 1.53, *p* = .137, ${\eta}_{p}^{2}=.11$.

### Discussion

This experiment investigated whether avatars created with our method can convey basic emotional expressions in a manner comparable to real human faces. All six expressions were recognized above chance and followed recognition patterns established in the emotion literature (Ekman, 1972; Elfenbein & Ambady, 2002; Izard, 1994). Specifically, angry, happy, and sad expressions were recognized with similar accuracy across avatars, UKF photographs, and the WFE. Disgusted expressions were classified equally well in avatars and UKF photographs, though less accurately than in the WFE. Surprise was recognized in avatars at levels comparable to the WFE but lower than UKF photographs. Fearful expressions, however, were consistently recognized less accurately in avatars compared to photographs, replicating previous findings that fear is among the most challenging emotions to convey and identify (Elfenbein & Ambady, 2002).

We also examined confusions between expressions and showed that these exhibit a similar pattern across tests, whereby common confusions typically occurred between disgusted and angry faces, and between fearful and surprised faces. This pattern matches those observed with images of faces (e.g.,Ekman, 1972; Izard, 1994). Finally, we also looked at correlations between tests in the classification of each expression. Most of these correlations failed to reach significance, likely due to ceiling effects in recognition performance and the small sample size. However, classification of fearful expressions was characterized by lower accuracy than the other expressions *and* by strong correlations between avatars and face photos. This points to a similar basis for classifying fearful expressions, despite the lower accuracy for this expression in avatars than in face photos.

Overall, these findings indicate that the avatars effectively convey dynamic emotional expressions. Although recognition of fear remains less accurate, the overall pattern of classification and confusions demonstrates that participants interpret the avatar expressions in a manner that is generally consistent with real human faces. This indicates that our method for equipping avatars with these expressions works well.

## General discussion

The present study sought to develop avatars capable of producing naturalistic eye gaze and facial expressions using an accessible and adaptable pipeline, enabling other researchers to implement these methods in their own work. We then carried out two experiments to validate these avatars as effective stimuli for behavioral research in virtual reality, by showing that the avatars elicit behavior consistent with findings from studies using real human faces. Experiment 1 shows that participants are faster to detect targets appearing in the direction of avatar gaze than in the opposite direction (e.g., Driver et al., 1999; Friesen & Kingstone, 1998). This effect emerged even with targets that appeared just 100 ms after a shift in eye gaze, indicating rapid and potentially reflexive attentional orienting. Experiment 2 then showed that avatars created through our pipeline also convey emotional expressions that participants can reliably recognize, with an overall pattern of classification and confusions that is highly consistent with the perception of emotional expressions from real human faces (Du & Martinez, 2013; Elfenbein & Ambady, 2002; Kohler et al., 2003; Thompson & Voyer, 2014).

These findings validate our avatar construction pipeline and highlight the utility of this method for researchers interested in conducting studies in virtual reality. Avatars offer significant advantages for such cognitive and behavioral research by acting as proxies for humans due to their ability to display complex social behaviors (Etienne et al., 2023; Garau et al., 2001; Geraets et al., 2021; Kimmel et al., 2023; Zheleva et al., 2023). Unlike human confederates, avatars can combine both experimental control and ecological validity by behaving in ways that are reproducible across trials and participants. Yet, avatars in prior studies have often lacked realism (e.g., Bülthoff et al., 2019; Tummon et al., 2019, 2020), raising questions about whether results generalize to interactions with real people. The realism of avatars may influence whether neural and behavioral responses align with those elicited by real humans (De Borst & De Gelder, 2015). Our construction pipeline makes progress on this issue by producing avatars that more closely approximate human appearance and behavior, helping to bridge this gap.

The potential applications of these avatars extend across domains. For example, an observer’s ability to embody an avatar is influenced by their visual resemblance (Kilteni et al., 2012; Suk & Laine, 2023). Our pipeline allows the generation of avatars that closely resemble participants’ faces, creating opportunities to investigate how realism and similarity affect embodiment. Moreover, real-time rigging enables user-controlled gaze and expression, opening avenues to study how agency over avatar facial movements modulates embodiment. Beyond embodiment, virtual avatars can enrich domains such as social psychology, for example, by enabling studies of interpersonal distance and approach–avoidance behavior through position tracking (Blascovich et al., 2002; Yaremych & Persky, 2019). Avatars could also help researchers explore questions on the presence of trusted partners in novel scenarios (Kane et al., 2012) or factors affecting visual perspective-taking (Ferguson et al., 2018). Avatars may also serve clinical applications, from assessing social cognition to delivering mental health interventions in controlled environments (Bell et al., 2020).

While our avatar construction technique provides an accessible method for creating photorealistic avatars capable of facial movement, improvements can be made. Facial motion is generated through 52 blend shapes. This is standard for using accessible face capture software such as that in the iPhone’s ARKIT, but offers more limited control over more complex facial movements such as those involved in speech. The use of template-based meshes further restricts the capture of individual idiosyncrasies in facial dynamics, which are known to aid face recognition (Burton et al., 2015; Mileva & Burton, 2018). While editing blend shapes could introduce greater individuality, this process requires specialist expertise in 3D modeling. Future work should explore methods for more accurately representing idiosyncratic motion patterns and test their influence on recognition and realism.

Finally, while the current validation experiments establish that our avatars elicit gaze following and emotion recognition, these behaviors were tested in relatively simple, decontextualized tasks. Yet, social context strongly shapes gaze perception (Frischen et al., 2007; Pfeiffer et al., 2013) and emotion interpretation (Barrett et al., 2011; Milanak & Berenbaum, 2014). Virtual reality provides the opportunity to embed avatars in rich, dynamic contexts to test how social and environmental factors interact with basic perceptual processes. The construction method described here offers a foundation for such work, equipping researchers with photorealistic, behaviorally valid avatars that can help bridge the divide between controlled laboratory studies and real-world social interactions.

## Supplementary Information

Below is the link to the electronic supplementary material.

Supplementary file1 (DOCX 2899 KB)

## Data Availability

Experiments 1 and 2 were pre-registered on the Open Science Framework and can be accessed at https://osf.io/jn3ky/ and https://osf.io/yjfzx/. The data for these experiments, the avatar construction pipeline and additional materials can be accessed at https://osf.io/xapwj/

## Footnotes

1 We use footnotes to explain key terms. An *avatar face mesh* is a 3D geometric model of a human face that is used to represent a person digitally. This mesh is constructed of vertices (points in 3D space) and edges (lines connecting the points). Within the face mesh, edges typically form triangles and quadrangles, which are referred to as polygons. A face mesh is therefore a polygonal surface that captures the shape (features, contours, proportions) of a human face. Face meshes can be based on real people and animated for movement.

2 The *topology* of an avatar (and other virtual objects) refers to its structural layout - that is, how the vertices, edges, and polygons are arranged. It also reflects the polygon count within this layout. A topology with many polygons can allow for more complicated animation and movement.

3 While topology captures the layout of a polygonal object, *geometry* refers to the actual 3D positions of its vertices. With regards to avatars, topology refers to how an avatar is built, and different avatars can follow the same topology, which is important for standardization in our production pipeline. In contrast, geometry refers to the actual shape of a specific avatar. Put differently, different avatar identities may share the same overall layout of the polygonal mesh, but the 3D position of each vertex (*x*, *y* and *z* coordinates) within this mesh may differ to encode differences between individuals (e.g., a long nose, high forehead, or wide jaw).

4 *Blend shapes* are predefined deformations of polygonal surfaces that systematically act on the *x*, *y*, and *z* coordinates of part of an avatar mesh. To animate avatar faces, some blend shapes may control different facial features (e.g., eyes, nose, mouth) or smaller regions within these features. Different blend shapes can be mixed together to create expressions on an avatar face.

5 We also analysed these data separately for the Bailenson et al. (2003) and Temple Presence Inventory social presence measures, with comparable results. A summary of these analyses can be accessed at https://osf.io/jn3ky/.
