# Supplementary material for: High-fidelity avatars for behavior and cognition research in virtual environments: An accessible construction method for scientists
Source: Behav Res Methods. 2026 Aug 19;58(9):275. doi: 10.3758/s13428-026-03139-1 (PMC13490262; doi:10.3758/s13428-026-03139-1)
Supplement: Supplementary file 1 [file 13428_2026_3139_MOESM1_ESM.docx]

**Appendix 1**

**Table 1**

*An outline of the steps involved in the avatar construction process.*

| Main step | Sub steps | Overview of process | Example |
| --- | --- | --- | --- |
| Scanning a face in 3D | - Load Artec Studio - Plug in Artec Eva 3D scanner into computer - Scan face of desired identity to create several scans - Fuse scans into a single mesh - Process scan to remove extraneous objects - Apply texture to scan - Export 3D scan and texture | The face of a real person, who is sat with a neutral expression, is scanned using the Artec Eva 3D scanner. The raw scans are then post-processed to make one coherent scan. |  |
| Wrapping the face mesh | - Install Faceform Wrap - Load wrapping pipeline and face mesh into Wrap - Align face mesh with template - Map facial landmarks on the face meshes - Compute the wrapping process - Refine wrapped mesh - Export wrapped mesh and texture | The topology of a template mesh is applied to the face scan using Faceform Wrap. This wrapped face scan is then exported for future steps. |  |
| Creating an avatar body | - Install Mixamo Fuse - Choose avatar head, torso, arms, and legs from presets in Mixamo Fuse - Make adjustments to avatar body dimensions - Add clothes to avatar body - Export avatar model and texture | An avatar body is created using Mixamo Fuse, a 3D character creator. Clothes are added once the body has been created. The full avatar, along with clothing, is then exported. |  |
| Combining the head and avatar body | - Install Autodesk Maya - Import the face scan and Mixamo Fuse avatar model into Maya - Remove the head from the Mixamo Fuse avatar - Remove access mesh from face scan and resize - Add texture to avatar head and avatar body - Adjust the UVs of textures - Combine face scan with Mixamo Fuse avatar body - Export combined mesh and textures | The face scan of the desired identity is combined with the avatar made in Mixamo Fuse. Before combining the two meshes, the layouts of the textures are adjusted to prevent overlap. |  |
| Wrapping the whole body | - Load the body wrap pipeline in Faceform Wrap - Insert combined avatar mesh and texture - Map landmarks between template mesh and combined avatar - Compute the wrapping process - Refine the wrapped mesh - Select the face polygons to export the face texture - Select the body polygons to export the body texture - Export the wrapped body mesh | The combined avatar is inserted back into Faceform Wrap to apply the topology of a template mesh. The wrapped mesh is exported along with separate texture files for the head and rest of the body. |  |
| Adjusting the avatar’s texture | - Install GIMP - Insert the face and body textures as layers - Remove excess texture from the face layer - Fix artifacts in the face scan texture - Adjust colour and lighting of the face and body texture to better match - Blend together the seams between the body and the face texture - Export the combined texture as a single file - Apply the combined texture to the avatar | The two separate textures from the face scan and avatar body are inserted into a single file in GIMP. Artifacts in the face texture are fixed and the two different textures are blended together. |  |
| Rigging the avatar in Mixamo | - Export the avatar as an Fbx file - Upload the avatar to Mixamo.com - Apply markers to the avatar to highlight the chin, elbows, wrists, groin, and knees - Wait for the auto-rigging process to complete - Apply a T-Pose animation - Export the rigged avatar | The avatar is imported into Mixamo, a website that has an auto-rigging process. Once the auto-rigging process is complete, the avatar has a skeleton that can be moved. |  |
| Setting up blend shapes | - Insert rigged avatar into Maya - Import template blend shapes - Apply blend shapes from template onto rigged avatar - Bake transferred blend shapes to rigged avatar | Blend shapes – deformations in the face mesh – are transferred from a template onto the rigged avatar. This process allows for facial animation. |  |
| Inserting teeth and eyes | - Insert eye and teeth meshes - Position eyes and teeth in the face mesh - Customise the teeth - Create four blend shapes for each eye – look in, look out, look up, look down - Create a blend shape for the lower teeth – teeth open - Connect eye movement to eyelid movement - Connect teeth movement to jaw movement | Eyes and teeth are inserted into the avatar. Blend shapes are created for the eyes to account for different gaze directions. A blend shape is created for the lower teeth to account for the jaw opening. |  |
| Creating eyelashes | - Duplicate the eyelids to create a base for the eyelashes - Use XGen in Maya to create guides for the eyelashes - Adjust the length and shape of the guides - Generate the eyelashes - Add a taper to the end of the lashes - Add noise to the lashes - Convert the generated lashes into polygons | Using XGen in Maya, eyelashes are created on the eyelids of the avatar. These eyelashes follow guides created by the user, so can be customised in terms of length and shape. |  |
| Rigging the eyelashes | - Select parts of the eyelids on the avatar mesh - Add rivets to these selected points - Add joints to the rivets - Bind the eyelashes to the joints - Create eyelash blend shapes - Remove rigging from eyelashes - Connect eyelash blend shapes to eye and face blend shapes | The eyelashes are rigged onto the eyelids to allow for eyelash blend shapes to be created. These eyelash blend shapes are then connected to eye and face blend shapes. |  |
| Creating facial expressions | - Install Rokoko Studio on computer - Install Rokoko Face Capture App on iPhone - Record facial expression on face capture app - Apply facial expression to avatar in Maya using the Rokoko Studio Plugin - Refine the facial expression using the graph editor or the AnimBot plugin | Facial expressions are recorded from a real person’s face on the Rokoko Face Capture app. The facial animation is then applied to the avatar using the Rokoko Studio Plugin in Maya. Refinements are made to the animation. |  |

**Appendix 2**

**Table 2.1**

*Items and corresponding scales in Bailenson et al.’s (2003) measure of social presence.*

| Scale | Items |
| --- | --- |
| -3 (*Strongly Disagree*)  to 3 (*Strongly Agree*) | 1. I perceive that I am in the presence of another person in the room with me. |
|  | 2. I feel that the person is watching me and is aware of my presence |
|  | 3. The thought that the person is not a real person crosses my mind often. |
|  | 4. The person appears to be sentient, conscious, and alive to me. |
|  | 5. I perceive the person as being only a computerized image, not as a real person. |
| -3 (*Not at all*) to 3 (*Very much*) | 6. How much did you like the virtual humans? |

**Table 2.2**

*Selected subscales, items and corresponding scales from the Temple Presence Inventory (Lombard et al., 2009).*

| Subscale | Scale | Item |
| --- | --- | --- |
| *Social Presence*  – parasocial interaction (actor within medium) | 1 (*Never*)  to 7 (*Always*) | 1. How often did you have the sensation that people you saw/heard could also see/hear you? |
|  | 1 (*None*)  to 7 (*Very much*) | 2. To what extent did you feel you could interact with the person or people you saw/heard? |
|  | 1 (*Not at all*)  to 7 (*Very much*) | 3. How much did it seem as if you and the people you saw/heard both left the places where you were and went to a new place? |
|  |  | 4. How much did it seem as if you and the people you saw/heard were together in the same place? |
|  | 1 (*Never*)  to 7 (*Always*) | 5. How often did it feel as if someone you saw/heard in the environment was talking directly to you? |
|  |  | 6. How often did you want to or did you make eye-contact with someone you saw/heard? |
|  | 1 (*Not at all*)  to 7 (*Very much*) | 7. Seeing and hearing a person through a medium constitutes an interaction with him or her. How much control over the interaction with the person or people you saw/heard did you feel you had? |
| *Social presence*  – passive interpersonal | 1 (*Not well*)  to 7 (*Very well*) | 8. During the media experience how well were you able to observe the facial expressions of the people you saw/heard? |
|  |  | 9. During the media experience how well were you able to observe the changes in tone of voice of the people you saw/heard? |
|  |  | 10. During the media experience how well were you able to observe the style of dress of the people you saw/heard? |
|  |  | 11. During the media experience how well were you able to observe the body language of the people you saw/heard? |
| *Social presence*  – active interpersonal | 1 (*Never*)  to 7 (*Always*) | 12. How often did you make a sound out loud (e.g. laugh or speak) in response to someone you saw/heard in the media environment? |
|  |  | 13. How often did you smile in response to someone you saw/heard in the media environment? |
|  |  | 14. How often did you want to or did you speak to a person you saw/heard in the media environment? |

**Table 2.3**

*Items and corresponding subscales from the User Engagement Scale – Long Form (O’Brien et al., 2018). Scales range from 1 ‘Strongly disagree’ to 7 ‘Strongly agree’ with 3 being ‘Neither agree not disagree’.*

| Subscale | Item |
| --- | --- |
| *Focus Attention* | 1. I lost myself in this experience. |
|  | 2. I was so involved in this experience that I lost track of time. |
|  | 3. I blocked things around me when I was using the application. |
|  | 4. When I was using the virtual room, I lost track of the world around me. |
|  | 5. The time I spent in the virtual room with the avatar just slipped away. |
|  | 6. I was absorbed in this experience. |
|  | 7. During this experience I let myself go. |
| *Perceived Usability* | 8. I felt frustrated while in the virtual room. |
|  | 9. I found this application confusing to use. |
|  | 10. I felt annoyed while using the application. |
|  | 11. I felt discouraged while using this application. |
|  | 12. Using this application was taxing. |
|  | 13. This experience was demanding. |
|  | 14. I felt in control while in the virtual room. |
|  | 15. I could not do some of the things I needed to do while in the virtual room. |
| *Aesthetic Appeal* | 16. This virtual room was attractive. |
|  | 17. This virtual room was aesthetically appealing. |
|  | 18. I liked the graphics and images of the virtual room. |
|  | 19. The virtual room appealed to my visual senses. |
|  | 20. The layout of the virtual room was visually pleasing. |
| *Reward* | 21. Using the application was worthwhile. |
|  | 22. I consider my experience a success. |
|  | 23. This experience did not work out the way I had planned. |
|  | 24. My experience was rewarding. |
|  | 25. I would recommend this experience to my family and friends. |
|  | 26. I continued to use the virtual room out of curiosity. |
|  | 27. The content of the experience incited my curiosity. |
|  | 28. I was really drawn into this experience. |
|  | 29. I felt involved in this experience. |
|  | 30. This experience was fun. |

**Appendix 3**

A 3 (test: avatar, University of Kent Faces, Warsaw Facial Expressions) × 6 (facial expression: happy, sad, angry, fearful, surprised, disgusted) within-subjects ANOVA was conducted on the accuracy data in Experiment 2. The following table displays Bonferroni-adjusted significance values, t-tests values and Cohen’s d effect sizes for comparisons across facial expressions (Table 3.1) and across tests (Table 3.2).

**Table 3.1**

*Bonferroni-adjusted significance values, t-test values and Cohen’s d effect sizes comparing accuracy across facial expressions in Experiment 2*.

|  |  |  | VR | | | UKF | | | | WFE | | |
| --- | --- | --- | --- | --- | --- | --- | --- | --- | --- | --- | --- | --- |
|  |  |  | *t*(27) | *p* | *d* | | *t*(27) | *p* | *d* | *t*(27) | *p* | *d* |
| angry | vs. | disgusted | 1.26 | 1.00 | 0.35 | | 3.43 | .296 | 0.67 | 1.42 | 1.00 | 0.13 |
|  | vs. | fearful | 1.68 | 1.00 | 0.78 | | 0.24 | 1.00 | 0.08 | 0.21 | 1.00 | 0.03 |
|  | vs. | happy | 6.21 | < .001 | 1.89 | | 6.67 | < .001 | 1.70 | 5.72 | < .001 | 0.74 |
|  | vs. | sad | 3.10 | .692 | 1.17 | | 5.63 | < .001 | 1.32 | 5.23 | .002 | 0.66 |
|  | vs. | surprised | 1.17 | 1.00 | 0.41 | | 3.90 | .089 | 1.02 | 0.26 | 1.00 | 0.04 |
| disgusted | vs. | fearful | 1.03 | 1.00 | 0.42 | | 2.34 | 1.00 | 0.59 | 0.66 | 1.00 | 0.10 |
|  | vs. | happy | 6.37 | < .001 | 2.25 | | 6.65 | < .001 | 1.04 | 6.51 | < .001 | 0.61 |
|  | vs. | sad | 3.85 | .100 | 1.53 | | 4.50 | .018 | 0.65 | 5.11 | .003 | 0.53 |
|  | vs. | surprised | 1.79 | 1.00 | 0.77 | | 2.27 | 1.00 | 0.36 | 0.87 | 1.00 | 0.09 |
| fearful | vs. | happy | 9.86 | < .001 | 2.67 | | 5.31 | .002 | 1.63 | 4.41 | .023 | 0.70 |
|  | vs. | sad | 7.36 | < .001 | 1.95 | | 4.15 | .046 | 1.24 | 4.31 | .030 | 0.62 |
|  | vs. | surprised | 3.65 | .171 | 1.19 | | 3.68 | .155 | 0.95 | 0.03 | 1.00 | 0.01 |
| happy | vs. | sad | 3.70 | .150 | 0.72 | | 4.35 | .027 | 0.38 | 2.18 | 1.00 | 0.08 |
|  | vs. | surprised | 10.17 | < .001 | 1.48 | | 6.00 | < .001 | 0.68 | 4.59 | .014 | 0.70 |
| sad | vs. | surprised | 3.05 | .782 | 0.76 | | 2.28 | 1.00 | 0.30 | 3.77 | .124 | 0.62 |

**Table 3.2**

Bonferroni-adjusted significance values, t-test values and Cohen’s d effect sizes comparing accuracy across tests in Experiment 2.

|  | VR vs. UKF | | | VR vs. WFE | | | UKF vs. WFE | | |
| --- | --- | --- | --- | --- | --- | --- | --- | --- | --- |
|  | *t*(27) | *p* | *d* | *t*(27) | *p* | *d* | *t*(27) | *p* | *d* |
| angry | 0.49 | 1.00 | 0.20 | 3.59 | .199 | 1.16 | 3.80 | .116 | 0.97 |
| disgusted | 3.36 | .355 | 1.22 | 4.84 | .007 | 1.65 | 3.01 | .862 | 0.43 |
| fearful | 4.99 | .005 | 1.05 | 9.59 | < .001 | 1.97 | 4.56 | .015 | 0.92 |
| happy | 0.57 | 1.00 | 0.01 | 0.57 | 1.00 | 0.01 | 0.00 | 1.00 | 0.00 |
| sad | 1.59 | 1.00 | 0.34 | 3.31 | .409 | 0.64 | 3.22 | .505 | 0.30 |
| surprised | 4.43 | .021 | 0.81 | 3.42 | .308 | 0.79 | 0.12 | 1.00 | 0.02 |
